# Long‐term on‐farm participatory maize breeding by stratified mass selection retains molecular diversity while improving agronomic performance

**DOI:** 10.1111/eva.12549

**Published:** 2017-10-14

**Authors:** Mara Lisa Alves, Maria Belo, Bruna Carbas, Cláudia Brites, Manuel Paulo, Pedro Mendes‐Moreira, Carla Brites, Maria do Rosário Bronze, Zlatko Šatović, Maria Carlota Vaz Patto

**Affiliations:** ^1^ Instituto de Tecnologia Química e Biológica António Xavier Universidade Nova de Lisboa Oeiras Portugal; ^2^ Instituto Nacional de Investigação Agrária e Veterinária Oeiras Portugal; ^3^ Departamento de Ciências Agronómicas Escola Superior Agrária de Coimbra Coimbra Portugal; ^4^ Faculdade de Farmácia Universidade de Lisboa Lisboa Portugal; ^5^ Instituto de Biologia Experimental e Tecnológica Oeiras Portugal; ^6^ Faculty of Agriculture Department of Seed Science and Technology University of Zagreb Zagreb Croatia

**Keywords:** ear traits, microsatellites, molecular diversity, on‐farm conservation, open‐pollinated populations, participatory plant breeding, yield, *Zea mays* L

## Abstract

Modern maize breeding programs gave rise to genetically uniform varieties that can affect maize's capacity to cope with increasing climate unpredictability. Maize populations, genetically more heterogeneous, can evolve and better adapt to a broader range of edaphic–climatic conditions. These populations usually suffer from low yields; it is therefore desirable to improve their agronomic performance while maintaining their valuable diversity levels. With this objective, a long‐term participatory breeding/on‐farm conservation program was established in Portugal. In this program, maize populations were subject to stratified mass selection. This work aimed to estimate the effect of on‐farm stratified mass selection on the agronomic performance, quality, and molecular diversity of two historical maize populations. Multilocation field trials, comparing the initial populations with the derived selection cycles, showed that this selection methodology led to agronomic improvement for one of the populations. The molecular diversity analysis, using microsatellites, revealed that overall genetic diversity in both populations was maintained throughout selection. The comparison of quality parameters between the initial populations and the derived selection cycles was made using kernel from a common‐garden experiment. This analysis showed that the majority of the quality traits evaluated progressed erratically over time. In conclusion, this breeding approach, through simple and low‐cost methodologies, proved to be an alternative strategy for genetic resources’ on‐farm conservation.

## 1 INTRODUCTION

Climate change represents a challenge to food security (Wheeler & von Braun, 2013). The negative impact of climate change on agriculture and therefore on food production is exacerbated by greater crop uniformity (Ceccarelli et al., 2010). An increasing number of studies show that biodiversity improves the capacity of agroecosystems to cope with extreme weather events and climate variability (Khoury et al., 2014; Ortiz, 2011), allowing crops’ evolution and adaptation to specific edaphic–climatic conditions (Ceccarelli, 2015). This is particularly important in the context of low‐input/organic production systems, more prone to biotic and abiotic constrains and in which crop resilience is fundamental. The greater uniformity of crops is specifically a concern for maize, wheat, and rice, which alone provide 60% of the calories in the human diet. In these three crops, recent plant breeding has led to extreme genetic uniformity (Ceccarelli, Galie, & Grando, 2013). As reviewed by Hellin, Bellon, and Hearne (2014), it is important that plant breeding reach a compromise by developing not only higher‐yielding but also stress‐tolerant cultivars, to allow them to cope and adapt when faced with different environmental conditions. In the case of maize, the more heterogeneous open‐pollinated populations, adapted to specific environmental conditions and human uses, have progressively been replaced in the last century by homogeneous, higher‐yielding commercial hybrids (Pingali, 2001). Still, open‐pollinated populations cultivation has been maintained, often in marginal lands or low‐input systems where commercial hybrids are not well adapted (Vaz Patto et al., 2013). They may also be kept by their dietary or nutritional value, taste, or for the price premium they attract because of high‐quality traditional properties that compensate for lower yields (Jarvis, Hodgkin, Sthapit, Fadda, & Lopez‐Noriega, 2011).

Portugal was one of the first European countries to adopt maize and one of the few where historical maize populations can still be found under cultivation (Vaz Patto et al., 2013). The resilience of these maize populations in the Portuguese scenario can be partially explained by their technological quality in maize bread production (Vaz Patto et al., 2013). The Portuguese ethnic maize‐based bread, named *broa*, is highly accepted for its distinctive sensory characteristics (Carbas et al., 2016). This bread is traditionally manufactured using local maize populations and still plays an important economic and social role on Central and Northern rural communities of the country (Vaz Patto, Moreira, Carvalho, & Pego, 2007). *Broa* is traditionally made with more than 50% maize flour mixed with rye and/or wheat flour by a mainly empirical process (Brites, Trigo, Santos, Collar, & Rosell, 2010). This process normally involves the mixing of the sieved wholemeal maize flour, with hot water, rye and/or wheat flour (in a variable proportion), and yeast from leavened dough from late *broa*, acting as sourdough (Brites et al., 2010).

In what concerns *broa* bread quality, differences between the higher‐yielding dent hybrids and the hard endosperm Portuguese open‐pollinated populations have been recently determined (Carbas et al., 2016). In that work, it was shown that the *broa* produced with the hybrid dent varieties had higher specific volume. However, sensory analysis showed a preference for the maize bread made using Portuguese open‐pollinated populations due to better mouthfeel flavor and texture (Carbas et al., 2016). Parameters associated with aroma or flavor (e.g., volatile aldehydes; Klensporf & Jelén, 2005, and texture (e.g., viscosity parameters; Brites et al., 2010) can be important in assessing the product's quality and therefore need to be investigated. Additionally, bread nutritional value is another quality aspect with great importance. In recent years, consumption of particular foods and food products, rich in antioxidant compounds, has been associated with the prevention of modern lifestyle‐related degenerative disease (Liu, 2003). In that regard, maize displays a considerable natural variation for content and composition of antioxidant compounds such as carotenoids (Owens et al., 2014) and tocopherols (Lipka et al., 2013). However, little is known about the phytochemical profiles, antioxidant activity, or organoleptic quality of the different Portuguese maize open‐pollinated populations with high technological ability for bread production.

With the development of modern sustainable low‐input agriculture in industrialized countries, for economic and environmental reasons, emphasis has been placed on local adaptation, on preservation of genetic diversity, and on quality (Cleveland, Soleri, & Smith, 1999). Conventional plant breeding has been successful in favorable environments, but is less successful in traditional low‐input or organic farming systems with higher stress growing conditions, especially in small‐scale farms (Vaz Patto et al., 2013). Under this scenario, participatory plant breeding (PPB) programs are arising worldwide to meet the needs of farmers in low‐input and organic environments that are normally overlooked by conventional crop breeders (Vaz Patto et al., 2013).

Participatory plant breeding differs from conventional breeding mainly because of the active participation of other actors apart from breeders, such as farmers and/or consumers, in the breeding program. Those actors can assume an active role in the establishment of the breeding objectives and influence or actively participate in the breeding activities. In the case of on‐farm participatory breeding, the selection is made at the farmer's field, in a partnership between breeder and farmer, with the farmer establishing the breeding objectives (Vaz Patto et al., 2013). Taking into consideration the central role attributed to farmers on this breeding approach, their acceptance and enthusiasm while participating in the program has been identified as one of the key aspects for the success of on‐farm participatory plant breeding (Vaz Patto et al., 2013). This type of decentralized PPB improves breeding efficiency as it increases the ratio of the number of varieties adopted by farmers, as it is the farmer's choice to adopt those varieties into the program; it also increases traits’ response to selection, as selection is being made in the targeted environment (Ceccarelli, 2015).

In 2012, Ceccarelli, Al‐Yassin, Goldringer, Mendes‐Moreira, and Chable (2012) published the results of a survey on the previous major PPB experiences worldwide. Of the 22 active PPB programs presented in that report, three are in maize and are located in Portugal, China, and Nepal. The Portuguese participatory maize breeding program started in 1984 and initially had as its main objective the improvement of the agronomic performance of historical maize populations, functioning in parallel as a strategy for the on‐farm conservation of those plant genetic resources (Vaz Patto et al., 2013).

The methodologies implemented in every breeding program are dependent on the type of reproductive system of the crop. In naturally cross‐pollinated species, such as maize, improvement of open‐pollinated populations can be achieved by recurrent mass selection if the pollinations are controlled and/or by the use of stratified selection (Gardner, 1961). In the on‐farm breeding activities of the Portuguese maize participatory breeding program, as controlled pollinations are time‐consuming, the use of stratified mass selection has been the selected methodology. In mass selection, a fraction of individuals are visually selected to form the following generation. As for stratified mass selection, prior to the selection of individuals (mass selection) the field is first divided into smaller selection units (field stratification), minimizing the bias due to field heterogeneity. The differences among plants within field's sections are more likely to be due to genetic differences than to environmental effects (Hallauer, Carena, & Miranda Filho, 2010). Stratified mass selection has been shown in the past to be a useful methodology for improving several agronomic traits in maize, for example, for adapting exotic germplasm into breeding programs and target environments (Hallauer, 1999) or for yield improvement of open‐pollinated maize populations (Mendes‐Moreira, Pego, Vaz Patto, & Hallauer, 2008; Mendes‐Moreira et al., 2009; Smith, Castillo, & Gómez, 2001).

In the Portuguese maize participatory breeding program, breeding activities were intended to occur mainly at the farmer's field, with breeder and farmer working side by side. Firstly, the selection methodologies were demonstrated by the breeder at each farmer's field, and afterward, the farmer conducted the same selection methodologies in the other part of the field. In this way, the farmer had a permanent possibility to compare the effectiveness of the breeder's advices and the breeder needed to respect the farmer's management system (e.g., low‐input), advising only simple and low‐cost selection methodologies based on population genetics theory, with the farmer keeping the decision power over the direction of selection. Besides the specific breeding objectives defined by each farmer for each maize population, in this program the farmer is advised by the breeder to select in the field by detasseling the undesirable plants before pollination (weakest and all that do not fit the desired ideotype, such as the pest and disease susceptible looking ones); the farmer is also advised to evaluate a few days before harvest the root and stalk quality by foot‐kicking the plants at their base (at the first visible internodes). This also serves as an indirect measurement of pest tolerance, as the plant that does not resist the impact and breaks down is eliminated. Additionally, the farmer is advised to favor the selection of more prolific plants or the ones with a lower ear insertion if that trait is among the farmer desired ideotype. Prior to this selection, the field is first divided into smaller selection units (field stratification). After harvesting, a second selection (postharvest) is conducted in the ears. This selection includes the specific breeding objectives of each population and the elimination of unhealthy damaged ears. Selected ears are then shelled and mixed together to form the next‐year generation. With this scheme, the selection pressure ranges from 1% to 5% (Mendes‐Moreira et al., 2009). Generally, the postharvest selection is the only selection that the farmer traditionally carries out (nonformal selection) and the one that had been applied to the historical maize populations previously to their introduction in this participatory program.

As recently reviewed by Fu (2015), besides aiming at the improvement of yield, adaptation, resistance to biotic and abiotic stresses, and end‐use quality, understanding and evaluating the impacts of (modern) plant breeding on crop genetic diversity is crucial to face the challenges of creating better crops/varieties capable of mitigating the constraints of fluctuating edaphic–climatic conditions. Moreover, genetic diversity studies can serve as a decision‐making tool for genetic resources’ management. This approach was applied, for example, in Lançon et al. (2008), in which the authors used molecular markers to access the genetic variability of cotton populations of a participatory breeding program and, as a direct result, farmers were advised to use another breeding methodology to increase the efficiency of the selection. Molecular markers have been also used to assess the temporal variation on maize genetic diversity due to human selection (Labate, Lamkey, Lee, & Woodman, 1999 and Solomon, Martin, & Zeppa, 2010). In Labate et al. (1999), the authors used molecular markers to study the effects of reciprocal recurrent selection on Iowa Stiff Stalk Synthetic and Iowa Corn Borer Synthetic maize populations, reporting a decrease in 39% of the mean expected heterozygosity after 12 cycles of selection. In Solomon et al. (2010), using other types of molecular markers to study the effects of reciprocal recurrent selection on tropical maize breeding populations, the authors reported a loss of 33%–37% of the alleles detected initially after 11 cycles of selection. Nevertheless, as reviewed by Rauf, Teixeira da Silva, Khan, and Naveed (2010), different plant breeding methods have shown different impacts on plant genetic diversity.

In the specific case of the Portuguese PPB program, the agronomic evaluation of the impact of breeding activities has only been performed in two of the several maize populations integrated in the program (Mendes‐Moreira et al., 2008, 2009), and the temporal changes on genetic diversity were only evaluated in one of those populations (Vaz Patto, Moreira, Almeida, Satovic, & Pego, 2008). Moreover, none of these studies took into consideration quality aspects that should be addressed in future breeding programs as the quality of these genetic resources for maize bread production seems to be a decisive aspect for the on‐farm maintenance of the historical populations developed (Brites et al., 2010; Vaz Patto et al., 2013).

The evaluation of the effect of stratified mass selection in the Portuguese maize participatory breeding program is crucial to understand whether the methodologies implemented in this program are effective or need to be revised in order to accomplish the defined breeding objectives. As a bulk of the harvested seed from each selection cycle was saved and kept in cold storage, it is possible to assess the evolution of the maize populations within the breeding program. Taking these factors into account, this work aimed to (i) evaluate whether on‐farm stratified mass selection, in the context of long‐term participatory research, was able to improve the agronomic performance of two historical maize open‐pollinated populations, Amiúdo and Castro Verde, (ii) evaluate the effect of stratified mass selection in the genetic diversity levels of the two populations, and (iii) evaluate the effect of stratified mass selection in quality traits (related to consumer preferences, technological, nutritional, and organoleptic properties) that may influence maize bread quality.

## 2 MATERIALS AND METHODS

### 2.1 Populations’ origin and main features

The two historical open‐pollinated maize populations evaluated in this study were previously subjected to on‐farm stratified mass selection in the context of a participatory breeding program. This breeding program has been running in Portugal since 1984 in the Sousa Valley region, in the northern part of the country. Each maize population in this breeding program occupied, on average, an area of 1,000 m^2^ and was composed of approximately 5,000 individuals per growing season (given a plant density of 50,000 plants/ha).

Amiúdo, a yellow flint early population (FAO 200), was chosen to integrate the PPB program in its beginning, in 1984. This population was selected due to its short life cycle and because it had already adapted to the local conditions (poor soils with low pH, water stress, and aluminum toxicity); it was also chosen because it could be used for bread production (Vaz Patto et al., 2013).

Amiúdo population was selected at two different locations: at the Lousada site (41°14′7.8″N 8°18′11.1″W), where the selection was performed by the breeder and farmer; and at the Serra do Carvalho site (41°34′12.74″N, 8°19′28.77″W), where the selection was performed by the breeder. In both cases, the specific breeding objective, set by the farmer, was to achieve a higher‐yielding population; the same selection methodologies were applied at both the Lousada and Serra do Carvalho sites.

Castro Verde, an orange flint late population (FAO 600), was introduced in the PPB program in 1994 with the initial aim of achieving a population that could run in the category of yellow flint in a contest for the “Best Ears” of the Sousa Valley. This population was characterized by its big ears and very tall plants (>3 m in height).

Until 2000, Castro Verde was selected at the Lousada site (41°14′7.8″N 8°18′11.1″W) by the farmer. The selection criteria were set to obtain bigger ears by improving the traits that might enable the ears to win the “Best Ears” contest, namely ear length and kernel weight, row number, and number of kernels per ear. After 2001, due to a reduction in the breeding activities at the Lousada site, the Castro Verde population began to be selected at the Coimbra site (40°13′0.22″N, 8°26′47.69″W) by the breeder. At that point, some adjustments were made to the breeding objectives but keeping the same selection methodologies (stratified mass selection). Specifically, selection criteria were fine‐tuned to decrease the height of the ear insertion on the stalk, increase the stalk resistance, and keep increasing the ear size while still maintaining an orange flint kernel.

As a result of 19 years of Amiúdo selection at Lousada site, 19 cycles of stratified mass selection were originated, and as a result of 25 years of Amiúdo selection at Serra do Carvalho site, 25 cycles of stratified mass selection were originated. In this study, the following Amiúdo cycles were analyzed: the initial population from 1984, considered as cycle 0 (hereafter referred to as AM_C0‐1984_), and the nineteenth and the twenty‐fifth cycles of stratified mass selection, obtained in 2003 at the Lousada site (hereafter referred to as AM‐L_C19‐2003_) and in 2009 at the Serra do Carvalho site (hereafter referred to as AM‐SC_C25‐2009_), respectively.

As a result of 14 years of Castro Verde selection, 14 cycles of stratified mass selection were originated between Lousada and Coimbra sites. In this study, the following Castro Verde cycles were analyzed: the initial population from 1994, considered as cycle 0 (hereafter referred to as CA_C0‐1994_), and the ninth and fourteenth cycles of stratified mass selection at Coimbra obtained in 2004 (hereafter referred to as CA‐C_C09‐2004_) and in 2009 (hereafter referred to as CA‐C_C14‐2009_), respectively.

**Figure 1:**
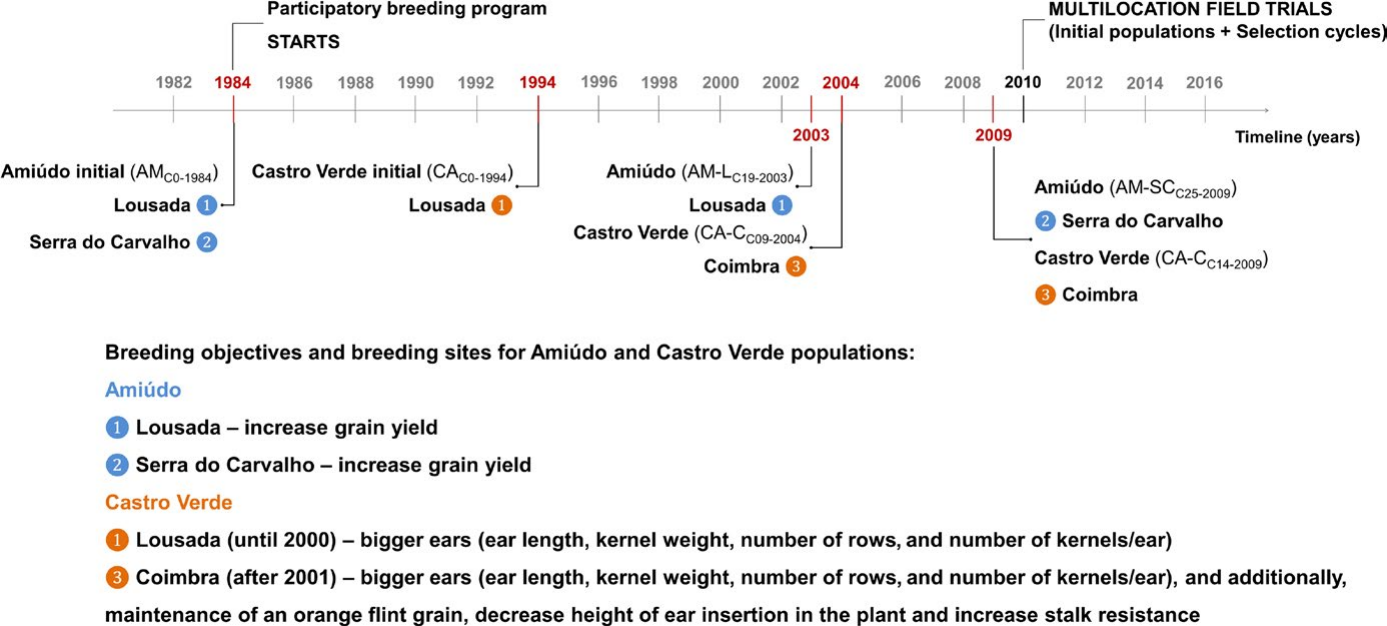
Breeding objectives, timeline, and selection sites for the analyzed Amiúdo cycles (initial population—AM_C__0‐1984_; AM‐L_C__19‐2003_ selection cycle; and AM‐SC_C__25‐2009_ selection cycle) and Castro Verde cycles (initial population—CA_C__0‐1994_; CA‐C_C__09‐2004_ selection cycle; and CA‐C_C__14‐2009_ selection cycle)

The summary of the specific breeding objectives for the Amiúdo and Castro Verde populations, as well as the timeline and selection sites where the different cycles, analyzed in this work, were developed, is given in Figure 1.

### 2.2 Agronomic evaluation

The agronomic performance of two historical maize populations, Amiúdo and Castro Verde, and their derived selection cycles was compared in multilocation field trials. The Amiúdo initial population (AM_C0‐1984_) and selection cycles (AM‐L_C19‐2003_ and AM‐SC_C25‐2009_) were evaluated in eight locations: Quinta da Conraria, Montemor‐o‐Velho, S. Pedro do Sul, Lousada, Valada do Ribatejo, Vouzela‐1, Vouzela‐2, and Travassos. The Castro Verde initial population (CA_C0‐1994_) and selection cycles (CA‐C_C09‐2004_ and CA‐C_C14‐2009_) were evaluated in five locations: Quinta da Conraria, Montemor‐o‐Velho, Lousada, Valada do Ribatejo, and Covas do Monte. The different locations represent different areas where maize open‐pollinated populations are traditionally produced in the country and also the different agronomic production systems normally associated with maize open‐pollinated populations, ranging from conventional production systems (Montemor‐o‐Velho) to organic production systems (Quinta da Conraria and Valada do Ribatejo) to low‐input production systems (all the other locations). Information about the sites’ characterization is given in Table S1. Initial populations and selection cycles were evaluated, at farmers’ fields, in a randomized complete block design, with three blocks per location. Each initial population and derived selection cycles were overplanted by hand in two‐row plots 6.4 m long and with 0.75 m between rows. Each plot was thinned at the seven‐leaf stage to 48 plants per plot to achieve a plant density of 50,000 plants/ha. Therefore, in each environment a total of 144 plants (48 plants per plot*3 blocks) were evaluated for each cycle. Plots were irrigated as needed and mechanically weeded and/or hand‐weeded as necessary. All the plots were harvested by hand.

The agronomic evaluation of each initial population and derived selection cycles was performed as described in Table 1. The data collected were intended to track eventual changes occurring in ear morphology, plant architecture, plant health and quality of the stalk and root system, population uniformity, and grain production.

**Table 1:** List of agronomic traits evaluated per plot basis, codes, and respective description

Type of trait	Trait	Code	Units/Scale	Description
Ear morphology	Ear weight	EW	Gram (g)	Ear weight, adjusted to 15% of grain moisture. Measured by averaging the weight of 4 shelled ears per plot.
	Cob weight	CW	Gram (g)	Cob weight, adjusted to 15% of grain moisture. Measured by averaging the weight of the cobs of 4 shelled ears per plot.
	Cob weight/ear weight	CWEW	Ratio (g/g)	Ratio cob/ear weight indicates the proportion of cob weight in the ear weight. This ratio was taken from the cob and ear weights of 4 shelled ears per plot.
	Ear moisture	EM	Percentage (%)	Measured with a FARMPOINT moisture meter, using a mixture sample of 4 shelled ears grain per plot.
Plant architecture	Ear placement	E	1–9 scale	Ear placement in the plant. In this scale, a 5 indicates that the first ear is located in the middle of the plant; and values <5 indicate that the first ear is located bellow the plant middle point; values >5 indicate that the first ear is located above the plant middle point. This value was measured by evaluating all plants per plot.
	Leaf angle	N	1–9 scale	Angle of the adaxial side of the leaf above the ear with the stalk. In this scale, a 5 indicates a leaf angle = 45 °; values <5 indicate a leaf angle <45°; and values > 5 indicate a leaf angle >45°. This value was measured by evaluating all plants per plot.
	Tassel branching	T	1–9 scale	In this scale, 1 indicates unbranched tassel (typical of inbred lines) and 9 indicates a highly branched tassel (frequent in populations with fasciated ears). This value was measured by evaluating all plants per plot.
Health and quality of the stalk and root system	Root lodging	R	Percentage (%)	Root lodging corresponds to percentage of plants leaning more than 30° from vertical in each plot. This value was measured by evaluating all plants per plot.
	Stalk lodging	S	Percentage (%)	Stalk lodging corresponds to percentage of plants broken at or below the primary ear node. This value was measured by evaluating all plants per plot.
	Standing plants	SP	No. plants/hectare (no. plants/ha)	Estimation of the number of standing plants per hectare given the number of plants at harvest time in the area of each plot (9.6 m^2^).
Population uniformity	Uniformity	U	1–9 scale	Measure of population uniformity. In this scale, 1 indicates minimum uniformity and 9 indicates maximum uniformity. Values from 1 to 4 are typical of open‐pollinated populations, and values from 5 to 9 are typical of pure lines. Measured by evaluating all plants per plot.
Grain production	Prolificacy	P	No. ears/plant	Total number of ears per plot divided by the total number of plants per plot.
	Grain yield	Y	Kilogram/hectare (kg/ha)	Grain yield adjusted to 15% moisture. Formula: Grain yield = Ear weight × (Grain weight/Ear weight) × (100%–% moisture at harvest)/(100%–15% moisture). Grain weight and ear weight taken from 4 shelled ears.
	Grain yield per plant	YP	Gram/plant (g/plant)	Grain yield adjusted to 15% moisture divided by the number of standing plants per hectare.

### 2.3 Agronomic data analysis

All agronomic data analysis was carried out in SAS software (SAS Release 9.2.; SAS Institute, 2004).

Analysis of variance for Amiúdo cycles (initial population—AM_C0‐1984_; AM‐L_C19‐2003_ selection cycle; and AM‐SC_C25‐2009_ selection cycle) and for Castro Verde cycles (initial population—CA_C0‐1994_; CA‐C_C09‐2004_ selection cycle; and CA‐C_C14‐2009_ selection cycle) was carried out separately per population using the PROC MIXED procedure. In the mixed‐model statement, environments and cycles (initial population and derived selection cycles) were treated as fixed effects, while blocks, treated as random, were nested in the environments. The interaction between cycles and the environment was included in the model. Cycle means were compared using a Tukey–Kramer multiple comparisons test.

To summarize multivariate changes occurring in both populations across the participatory breeding program, a principal component analysis (PCA) on the standardized agronomic data was performed using the PROC PRINCOMP procedure. The number of principal components was determined by inspecting eigenvalues of principal components (using the Kaiser criterion that retains components with eigenvalues greater than one). The first two principal components were then projected in a biplot to display shifts occurring in the agronomic traits measured on both initial populations and their selection cycles.

### 2.4 Molecular evaluation

Thirty random individual plants from the Amiúdo and Castro Verde initial populations and derived selection cycles were genotyped with 20 microsatellites (SSRs—simple sequence repeats). SSRs were chosen based on their location in the maize reference genome (1 SSR per chromosome arm) and repeat motifs (≥3 base pairs) to facilitate allele scoring (Table S2). Information about each SSR can be found at MaizeGDB (Lawrence et al., 2008, www.maizegdb.org).

DNA was isolated from adult leaves of each plant using the modified CTAB procedure as described in Saghai‐Maroof, Soliman, Jorgensen, and Allard (1984). DNA quality was accessed using a 0.8% SeaKem^®^ LE Agarose gel (Cambrex Bio Science Rockland, Inc., USA) stained with SYBR^®^ Safe (Invitrogen, USA). DNA quantification was performed using a spectrophotometer, Nanodrop ND‐2000C (Thermo Scientific, USA). An additional step for polysaccharide removal (Rether, Delmas, & Laouedj, 1993) was added when the ratio 260/230 nm wavelength was inferior to 1.6 to avoid the interference of these contaminants in SSR amplification.

The SSR loci were amplified using a nested‐PCR method (Schuelke, 2000). PCR products were separated on 6.5% polyacrylamide sequencing gel (20 μl 6.5% KB^Plus^ Gel Matrix, 150 μl APS 10%, and 15 μl TEMED) using a LI‐COR 4300 DNA analyzer system. To account for any variance between PCR amplifications and electrophoresis runs, DNA from the B73 maize inbred line was used as a reference sample. Scoring of the alleles was confirmed manually by two independent users to insure scoring accuracy. A genotypic matrix of the alleles per individual plant, scored in base pairs, was generated and served as the basis for the molecular data analysis.

### 2.5 Molecular data analysis

To assess the intracycle genetic diversity, the average number of alleles per locus (N_av_), observed (H_O_) and expected heterozygosity (H_E_), and inbreeding coefficient (F_IS_) were calculated for each initial population and selection cycles using GENEPOP software (GENEPOP v4.0; Raymond & Rousset, 1995). The values of these estimates, obtained in each initial population and selection cycles, were then compared to test whether the values of N_av_, H_O_, H_E_, and F_IS_ were significantly different among cycles with the Kruskal–Wallis test using SAS software (SAS Release 9.2, SAS Institute Inc 2004).

The genotypic frequencies for each locus and for each Amiúdo and Castro Verde cycles were tested for conformance to Hardy–Weinberg (HW) expectations using GENEPOP software (GENEPOP v4.0; Raymond & Rousset, 1995). The probability test was based on the Markov chain method (Guo & Thompson, 1992; Raymond & Rousset, 1995) using 10,000 dememorization steps, 20 batches, and 5,000 iterations per batch. The sequential Bonferroni adjustments (Rice, 1989) were then applied to correct for the effect of multiple tests using SAS software (SAS Release 9.2, SAS Institute Inc 2004).

Differences in allele frequencies distributions along the breeding program were tested according to Waples (1989a), in which the null hypothesis states that the observed differences in allele frequency can be explained entirely by genetic drift and sampling error. For the Amiúdo population, the temporal variation in allele frequencies was tested (i) between the Amiúdo initial population (AM_C0‐1984_) and the selection cycle from the Lousada site (AM‐L_C19‐2003_), and (ii) between the Amiúdo initial population (AM_C0‐1984_) and the selection cycle from the Serra do Carvalho site (AM‐SC_C25‐2009_). For the Castro Verde population, the temporal variation in allele frequencies was tested between the initial Castro Verde population (AM_C0‐1984_) and the latter selection cycle from the Coimbra site (CA‐C_C14‐2009_). Afterward, the sequential Bonferroni adjustments (Rice, 1989) were applied to the level of significance to correct for the effect of multiple tests using SAS software (SAS Release 9.2, SAS Institute Inc 2004). The effective population size, which is a parameter necessary to test for temporal variation in allele frequencies, according to Waples (1989a), was estimated using NeEstimator software (NeEstimator v2.01, Do et al., 2014) following the temporal‐based method under sample plan II (Waples, 1989b), as the samples analyzed did not return to the breeding program. Alleles with a frequency lower than 0.05 were excluded, parametric chi‐squared 95% confidence intervals for effective population size were calculated, and the variance in allele frequencies was calculated according to Nei and Tajima (1981).

Analysis of molecular variance (AMOVA; Excoffier, Smouse, & Quattro, 1992), which is a method of estimating population differentiation directly from molecular data, was used to test whether the different cycles from Amiúdo and Castro Verde populations had suffered genetic differentiation along the breeding program. This was done by testing the partition of the total microsatellite diversity between and within each pair of cycles, as well as among and within all cycles using ARLEQUIN software (ARLEQUIN v3.0; Excoffier, Laval, & Schneider, 2005). The variance components retrieved from AMOVA were used to calculate a series of statistics called ϕ‐statistics, which summarize the degree of differentiation between population divisions and are analogous to Wright's *F*‐statistics (Excoffier et al., 1992). The variance components were tested statistically by nonparametric randomization tests using 10,000 permutations in ARLEQUIN software (ARLEQUIN v3.0, Excoffier et al., 2005).

To represent genetic relationships among individual plants, a factorial correspondence analysis (FCA) was carried out using GENETIX software (GENETIX v4.05; Belkhir, Borsa, Chikhi, Raufaste, & Bonhomme, 2004), as this analysis provides a way of visually showing how genetically distant the different initial populations and derived selection cycles are; it also serves as a method for observing the level of genetic homogeneity within each cycle.

### 2.6 Quality evaluation

As both populations are used for human consumption, we also measured in each of the Amiúdo and Castro Verde initial populations and derived selection cycles several traits associated with kernel quality. Therefore, this study also intended to evaluate in which way traits related to flour's pasting behavior (flour viscosity parameters), nutritional value (protein, fat, and fiber content), potential bioactive compounds (carotenoids, tocopherols, total phenolic compounds content), and aroma‐related compounds (volatile aldehydes) have changed or were maintained along the PPB program. For that, a bulk of kernel from each selection cycle produced from a common‐garden experiment established in Coimbra in 2009, under controlled pollinations, was used.

Wholemeal maize flour was obtained after milling the kernel through a Falling number 3100 mill (Perten, Sweden), using a 0.8‐mm screen.

#### 2.6.1 Pasting behavior

The pasting properties of maize flour were obtained with a Rapid Viscosity Analyzer RVA‐4 (Newport Scientific, Australia) at 15% solids as described in Brites et al. (2010). Peak (PV), minimum or trough (TV), and final viscosities (FV) were recorded in cPoise, and the breakdown (BD) was calculated as PV‐TV.

#### 2.6.2 Flour color parameters

Maize flour color was determined on 10–12 g of sample in an opaque recipient using a Minolta chromameter CR‐2b and CIE tristimulus color parameters: L*—lightness; a*—red/green index; and b*—yellow/blue index. L* values can vary from L* = 0 (black) to L* = 100 (white); positive a* values mean that samples tend toward the red part of the color spectra; and positive b* values mean that samples tend toward the yellow part of the color spectra.

#### 2.6.3 Protein, fat, and fiber content

Flour protein (PR), fat (FT), and fiber (FI) content were determined by a near‐infrared spectroscopic method with an Inframatic 8620 equipment (Perten, Sweden), with calibrations supplied by the manufacturer. Results were expressed in percentage.

#### 2.6.4 Total carotenoid content

The total carotenoid content (TCC) was spectrophotometrically measured at 450 nm according to the AACCI method 14‐60.01 (AACC International 2012). Results were expressed in μg of lutein equivalent per gram of sample, as the main carotenoid found in maize.

#### 2.6.5 Tocopherols content

α‐Tocopherol (AT), γ‐tocopherol (GT), and δ‐tocopherol (DT) were separated from the fat portion of the maize flours by high‐performance liquid chromatography (HPLC) and quantified using an Agilent 1200 model with a fluorescence detector (FLD) and a Diol column (LiChropher 100, 250 × 4 mm) according to the method ISO 9936 (2006). Tocopherols content was expressed in μg/g fat basis.

#### 2.6.6 Total free phenolic content

Ethanolic extracts (EtOH:H_2_O 50:50, v/v) for assessing the total phenolic content (PH) of maize flour were prepared as described in Lopez‐Martinez et al. (2009), with some modifications as described in detail in Supplementary Material.

Total free phenolic content was assessed using the Folin–Ciocalteu assay (Singleton, Orthofer, & Lamuela‐Raventos, 1999) with a Beckman DU‐70 spectrophotometer, with slight modifications as described in Silva et al. (2015), and expressed in mg of gallic acid equivalents/100 g of dry weight (GAE/100 g DW).

#### 2.6.7 *p*‐Coumaric and ferulic acid content

*p*‐Coumaric (CU) and ferulic acid (FE) were quantified by HPLC coupled with a photodiode array detector (HPLC‐PDA) at 280 nm with a Thermo Finnigan Surveyor HPLC system according to Silva, Gomes, Leitão, Coelho, and Vilas Boas (2006). *p*‐Coumaric (CU) and ferulic acid contents were expressed in mg/100 g of dry weight (mg/100 g DW).

#### 2.6.8 Volatile aldehydes content

The volatile fraction of maize flour was analyzed by solid‐phase microextraction–gas chromatography–mass spectrometry (SPME‐GC‐MS). A 2‐cm 50/30‐μm DVB/Carboxen/PDMS fiber (SUPELCO) was used for solid‐phase microextraction. Volatile compounds were analyzed with a GCMS‐QP2010 Plus Shimadzu equipment and separated in a Varian Factor Four column (30 m × 0.25 mm × 0.25 μm). Volatile aldehydes content (AL) was taken as the sum of the peak area of the main aldehydes identified (hexanal, heptenal, 2‐heptanal (*Z*), 2‐octenal (*E*), nonanal, 2‐nonenal (*E*), and decanal). Details on the quantification of volatile aldehydes content can be found in Supplementary Material.

### 2.7 Quality data analysis

To summarize the eventual multivariate changes on the evaluated quality traits occurring in both populations across the participatory breeding program, a principal component analysis (PCA) was performed using the PROC PRINCOMP procedure after standardization of the quality traits, similar to what has been already described for the agronomic data analysis.

## 3 RESULTS

In this work, the agronomical, molecular, and quality evolution of two historical open‐pollinated maize populations, Amiúdo and Castro Verde, across a participatory plant breeding program was accessed.

### 3.1 Agronomic evolution

In relation to the Amiúdo population agronomic performance, on‐farm stratified mass selection led, in both selection sites—Lousada and Serra do Carvalho—to a significant increase in ear (EW) and cob weight (CW) and cob/ear weight ratio (CWEW) (0.9%–1.2% for EW, 2.1%–3% for CW, and 1%–1.6% gain per cycle for CWEW, respectively) as well as to a significant gain in grain yield per plant (0.9% gain per cycle) and in grain yield overall (0.8% gain per cycle) (Table 2). The Amiúdo selection cycle from the Lousada site also had a significant increase in the levels of ear moisture (0.5% gain per cycle) when compared with the initial population (Table 2). The selection performed at the Serra do Carvalho site gave rise to an Amiúdo population with a decreased percentage of stalk lodging (−1.4% gain per cycle), and to an increase in tassel branching (0.4% gain per cycle) (Table 2).

**Table 2:** Analysis of variance, comparison of mean values, and percentage of gain per selection cycle for the agronomic traits among Amiúdo initial population (AM_C0‐1984_) and selection cycles from Lousada (AM‐L_C19‐2003_) and Serra do Carvalho (AM‐SC_C25‐2009_)

Trait	Analysis of variance^a^			Comparison of initial population/selection cycle means^b^			% gain/cycle	% gain/cycle
	Cycle	Env	Cycle*Env	AM_C0‐1984_	AM‐L_C19‐2003_	AM‐SC_C25‐2009_	AM‐L_C19‐2003_	AM‐SC_C25‐2009_
Ear weight (EW), in g	***	ns	ns	124.35 b	146.07 a	162.56 a	0.9	1.2
Cob weight (CW), in g	***	*	ns	20.29 b	31.85 a	30.83 a	3.0	2.1
Cob weight/ear weight (CWEW), in g/g	***	***	ns	0.16 b	0.21 a	0.20 a	1.6	1.0
Ear moisture (EM), in %	*	***	ns	18.84 b	20.59 a	20.13 ab	0.5	—
Ear placement (E), in 1–9 scale^c^	ns	ns	ns	5.54 a	5.29 a	5.38 a	—	—
Leaf angle (N), in 1–9 scale^d^	ns	**	ns	5.42 a	5.25 a	5.29 a	—	—
Tassel branching (T), in 1–9 scale^e^	*	***	ns	6.21 a	6.44 ab	6.79 a	—	0.4
Root lodging (R), in %	ns	*	ns	5.48 a	7.29 a	6.32 a	—	—
Stalk lodging (S), in %	*	**	ns	9.81 a	9.53 a	6.30 b	—	−1.4
Standing plants (SP), in no. plants/ha	ns	**	ns	49236 a	50062 a	49996 a	—	—
Uniformity (U), in 1–9 scale^f^	ns	ns	ns	3.42 a	3.58 a	3.38 a	—	—
Prolificacy (P), in no. ears/plant	ns	ns	ns	1.07 a	1.10 a	1.05 a	—	—
Grain yield (Y), in kg/ha	**	ns	ns	4568.84 b	5322.79 a	5577.93 a	0.8	0.8
Grain yield per plant (YP), in g/plant	**	ns	ns	93.00 b	107.88 a	112.57 a	0.9	0.9

a Significance for analysis of variance among cycles (initial population plus selection cycles) and among environments (Env) and interaction between cycles and environments (Cycle*Env): ns—nonsignificant; *—significant at *p* < .05; **—significant at *p* < .01; ***—significant at *p* < .001.

b Tukey–Kramer multiple comparisons test—mean values in each row followed by the same letter are not significantly different at *p* < .05.

c Ear placement (E), in 1–9 scale: 5 indicates that the first ear is located in the middle of the plant; values <5 indicate that the first ear is located bellow the plant middle point; and values >5 indicate that the first ear is located above the plant middle point.

d Leaf angle (N), in 1–9 scale: 5 indicates a leaf angle = 45 °; values <5 indicate a leaf angle <45 °; and values >5 indicate a leaf angle >45 °.

e Tassel branching (T), in 1–9 scale: 1 indicates unbranched tassel and 9 indicates a highly branched tassel.

f Uniformity (U), in 1–9 scale: 1 indicates minimum uniformity and 9 indicates maximum uniformity.

In relation to the Castro Verde population, on‐farm stratified mass selection did not lead to any significant differences in the mean values of the agronomic traits evaluated in this work (Table 3). For both Amiúdo (Table 2) and Castro Verde (Table 3), no significant genotype x environment interaction was detected for the agronomic traits evaluated.

**Table 3:** Analysis of variance and comparison of mean values for the agronomic traits among Castro Verde initial population (CA_C0‐1994_) and selection cycles (CA‐C_C09‐2004_ and CA‐C_C14‐2009_)

Trait	Analysis of variance^a^			Comparison of initial population/selection cycle means^b^		
	Cycle	Env	Cycle*Env	CA_C0‐1994_	CA‐C_C09‐2004_	CA‐C_C14‐2009_
Ear weight (EW), in g	ns	ns	ns	240.12 a	256.46 a	247.22 a
Cob weight (CW), in g	ns	**	ns	57.93 a	65.79 a	58.12 a
Cob weight/ear weight (CWEW), in g/g	ns	***	ns	0.24 a	0.26 a	0.23 a
Ear moisture (EM), in %	ns	**	ns	24.20 a	24.81 a	24.20 a
Ear placement (E), in 1–9 scale^c^	ns	*	ns	6.00 a	6.40 a	6.03 a
Leaf angle (N), in 1–9 scale^d^	ns	ns	ns	5.13 a	5.15 a	4.87 a
Tassel branching (T), in 1–9 scale^e^	ns	ns	ns	7.07 a	7.14 a	6.97 a
Root lodging (R), in %	ns	**	ns	31.99 a	31.50 a	22.53 a
Stalk lodging (S), in %	ns	***	ns	25.20 a	25.22 a	27.93 a
Standing plants (SP), in no. plants/ha	ns	**	ns	48,924 a	47,100 a	48,403 a
Uniformity (U), in 1–9 scale^f^	ns	**	ns	3.77 a	3.80 a	3.63 a
Prolificacy (P), in no. ears/plant	ns	ns	ns	0.98 a	1.00 a	0.90 a
Grain yield (Y), in kg/ha	ns	*	ns	6,862.71 a	6,851.03 a	6,840.93 a
Grain yield per plant (YP), in g/plant	ns	ns	ns	146.33 a	147.15 a	144.52 a

a Significance for analysis of variance among cycles (initial population plus selection cycles) and among environments (Env) and interaction between cycles and environments (Cycle*Env): ns—nonsignificant; *—significant at *p* < .05; **—significant at *p* < .01; ***—significant at *p* < .001.

b Tukey–Kramer multiple comparisons test—mean values in each row followed by the same letter are not significantly different at *p* < .05.

c Ear placement (E), in 1–9 scale: 5 indicates that the first ear is located in the middle of the plant; values < 5 indicate that the first ear is located bellow the plant middle point; and values > 5 indicate that the first ear is located above the plant middle point.

d Leaf angle (N), in 1–9 scale: 5 indicates a leaf angle = 45 °; values < 5 indicate a leaf angle <45 °; and values > 5 indicate a leaf angle > 45 °.

e Tassel branching (T), in 1–9 scale: 1 indicates unbranched tassel and 9 indicates a highly branched tassel.

f Uniformity (U), in 1–9 scale: 1 indicates minimum uniformity and 9 indicates maximum uniformity.

**Figure 2:**
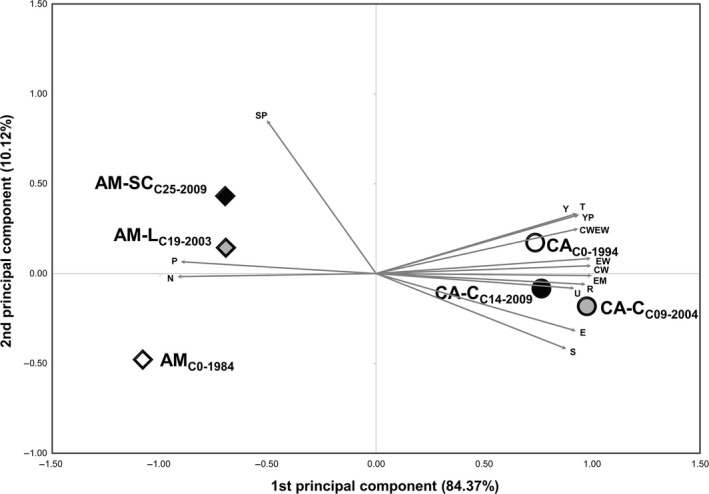
Biplot of principal component analysis (PCA) based on 14 agronomic traits measured in the Amiúdo cycles (initial population—AM_C__0‐1984_; AM‐L_C__19‐2003_ selection cycle; and AM‐SC_C__25‐2009_ selection cycle) and Castro Verde cycles (initial population—CA_C__0‐1994_; CA‐C_C__09‐2004_ selection cycle; and CA‐C_C__14‐2009_ selection cycle)

A principal component analysis based on the agronomic data was used to summarize the multivariate changes occurring in both populations across the participatory breeding program. The first two principal components for both the Amiúdo and Castro Verde cycles retained 94.49% of the total variance, with the first component already retaining 84.37% of observed variance (Figure 2). In the PCA biplot (Figure 2), the first axis clearly separated the Amiúdo from the Castro Verde populations. Moreover, for Amiúdo the first axis separated the initial population (AM_C0‐1984_) from the two selection cycles (AM‐L_C19‐2003_ and AM‐SC_C25‐2009_) in the direction of an increase in all the traits analyzed except for plant prolificacy (P) and the angle of the leaf insertion in the stalk (N) that decreased in this direction. The second axis separated the two selection cycles, AM‐L_C19‐2003_ and AM‐SC_C25‐2009_, in the direction of an increase in the number of plants standing (SP), with the selection cycle from the Serra do Carvalho site having a higher number of plants standing. As for Castro Verde, and as expected by the results obtained previously for the analysis of variance (Table 3), no clear progression was observed along the selection process when comparing the position on the biplot of the initial population CA_C0‐1994_, the cycle from 2004 (CA‐C_C09‐2004_), and the cycle from 2009 (CA‐C_C14‐2009_) (Figure 2).

### 3.2 Molecular diversity evolution

#### 3.2.1 Intrapopulation diversity

The molecular diversity analysis allowed tracing the overall genetic diversity evolution in the two open‐pollinated populations under study. In terms of quantitative differences in the alleles detected for the Amiúdo population, 73.26% of all alleles were maintained throughout the cycles: Of the 86 alleles detected, 63 were common to all the cycles (Table S3). Only six to eight alleles (7%–9.3%), out of the 74 identified in the initial population (AM_C0‐1984_), were not detected in the Serra do Carvalho (AM‐SC_C25‐2009_) and in the Lousada (AM‐L_C19‐2003_) selection cycles, respectively (Table S2). Likewise, in terms of quantitative differences in the alleles detected for Castro Verde population, the majority of the alleles (65.91%) were maintained throughout the cycles: Of 88 alleles detected, 58 were common to all the cycles (Table S3). Only 10 alleles (11.4%), out of the 74 detected in the initial population, were not detected in the CA‐C_C14‐2009_ selection cycle (Table S2).

As for the allelic frequencies, for both Amiúdo and Castro Verde populations a considerable proportion of the alleles detected were present in low frequencies (0.1 or less): Amiúdo cycles with 39.19% at the initial population (AM_C0‐1984_), 41.89% at the selection cycle from the Lousada site (AM‐L_C19‐2003_), and 48.10% at the selection cycle from the Serra do Carvalho site (Fig. S1A); and Castro Verde cycles with 47.30% at initial population (CA_C0‐1994_), 48.61% at the CA‐C_C09‐2004_ selection cycle, and 50% at the CA‐C_C14‐2009_ selection cycle (Fig. S1B).

When testing for significant differences among cycles within each population in the average number of alleles detected, observed and expected heterozygosity, and inbreeding coefficients, no significant differences were observed among the cycles for both the Amiúdo and Castro Verde populations (Table 4).

**Table 4:** Genetic variability estimates for Amiúdo initial population (AM_C0‐1984_) and Castro Verde initial population (CA_C0‐1994_) and derived selection cycles

Population/Selection cycle	*N*	*N*_av_	*N*_pr_	H_O_	H_E_	*F*_IS_	*p*‐Value HWE
AM_C0‐1984_	30	3.70	3	0.537	0.532	−0.009	ns
AM‐L_C19‐2003_	30	3.70	1	0.523	0.531	0.014	ns
AM‐SC_C25‐2009_	30	3.95	4	0.503	0.526	0.042	**
*p*‐value^a^ (KW)		.961		.584	.725	.520	
CA_C0‐1994_	30	3.70	4	0.482	0.482	0.000	ns
CA‐C_C09‐2004_	30	3.60	2	0.456	0.482	0.054	ns
CA‐C_C14‐2009_	30	3.80	6	0.457	0.498	0.082	*
*p*‐value^a^ (KW)		.911		.790	.930	.825	

*N*, number of individuals; *N*_av_, average number of alleles; *N*_pr_, number of private alleles; H_O_, observed heterozygosity; H_E_, gene diversity or expected heterozygosity; *F*_IS_, inbreeding coefficient; *p*‐value HWE, The probability global test for Hardy–Weinberg equilibrium (HWE) for each cycle was based on Markov chain method; ns, nonsignificant.

^a^ *p*‐Value of Kruskal–Wallis test among cycles (initial populations and derived selection cycles).

*Significant at *p* < .05; **Significant at *p* < .01; ***Significant at *p* < .001.

The global Hardy–Weinberg equilibrium test detected a significant departure from Hardy–Weinberg equilibrium in the Amiúdo cycle, AM‐SC_C25‐2009_, and in the Castro Verde cycle, CA‐C_C14‐2009_, both due to heterozygote deficiency (*F*_IS_ = 0.042, *p*‐value <.01; and *F*_IS_ = 0.082, *p*‐value <.05, respectively) (Table 4). When testing for the departure from Hardy–Weinberg equilibrium by individual locus in both the Amiúdo and Castro Verde populations, the majority of the loci had their genotypic frequencies in accordance with Hardy–Weinberg expectations (Table S4).

With the objective of testing for temporal changes in the allele frequencies distribution, the effective population size (*N*_e_) was estimated by a temporal‐based method under sample plan II. For Amiúdo, the estimated effective population size for the Lousada site was *N*_e_ = 119.6, while for the Serra do Carvalho site the *N*_e_ value was bigger (*N*_e_ = 243.7) (Table S5). For Castro Verde, the estimated effective population size was *N*_e_ = 161.7 (Table S5). After a Bonferroni multiple‐test correction, no significant temporal variation of allele frequencies was detected for both populations and selection sites (Amiúdo: Table S6; Castro Verde: Table S7).

#### 3.2.2 Differentiation among cycles

The genetic differentiation among cycles within each population was tested following the framework of AMOVA. The AMOVA results showed that for the Amiúdo population, the percentage of variance that could be attributed to differences among all cycles represented 2.86% of the total molecular variation (Table 5). The pairwise comparisons between Amiúdo cycles showed that stratified mass selection led overall to a significant but small genetic differentiation (given the significant ϕ_ST_ values; Table 5). For the Castro Verde population, AMOVA showed that the variation among all cycles represented only 1.72% of the total molecular variation (Table 5). In this case, stratified mass selection did not generate a significant genetic differentiation between CA_C0‐1994_ and CA‐C_C09‐2004_ (ϕ_ST_ = 0.003, *p*‐value >.05) (Table 5).

**Table 5:** Analysis of molecular variance (AMOVA) results for the partitioning of SSR variation among and within Amiúdo cycles (AM_C0‐1984_, AM‐L_C19‐2003_, and AM‐SC_C25‐2009_) and Castro Verde cycles (CA_C0‐1994_, CA‐C_C09‐2004_, and CA‐C_C14‐2009_)

Comparison	% Total variance		ϕ‐statistics^a^	*p* (ϕ)^b^
	Among cycles	Within cycles		
AM_C0‐1984_ vs. AM‐L_C19‐2003_	4.33	95.67	0.043	***
AM_C0‐1984_ vs. AM‐SC_C25‐2009_	2.98	97.02	0.030	***
AM‐L_C19‐2003_ vs. AM‐SC_C25‐2009_	1.22	98.78	0.012	*
All Amiúdo cycles	2.86	97.14	0.029	***
CA_C0‐1994_ vs. CA‐C_C09‐2004_	0.34	99.66	0.003	ns
CA_C0‐1994_ vs. CA‐C_C14‐2009_	2.40	97.60	0.024	***
CA‐C_C09‐2004_ vs. CA‐C_C14‐2009_	2.36	97.64	0.024	***
All Castro Verde cycles	1.72	98.28	0.017	***

^a^ϕ‐statistics: corresponding to an analogous to the fixation index (F_ST_) which measures the degree of genetic differentiation among populations/selection cycles (ϕ_ST_).

^b^ *p*(ϕ): The level of significance of the ϕ‐statistics was tested by nonparametric randomization tests using 10,000 permutations.

ns, nonsignificant; *Significant at *p* < .05; **Significant at *p* < .01; ***Significant at *p* < .001.

#### 3.2.3 Genetic relationships among individuals

**Figure 3:**
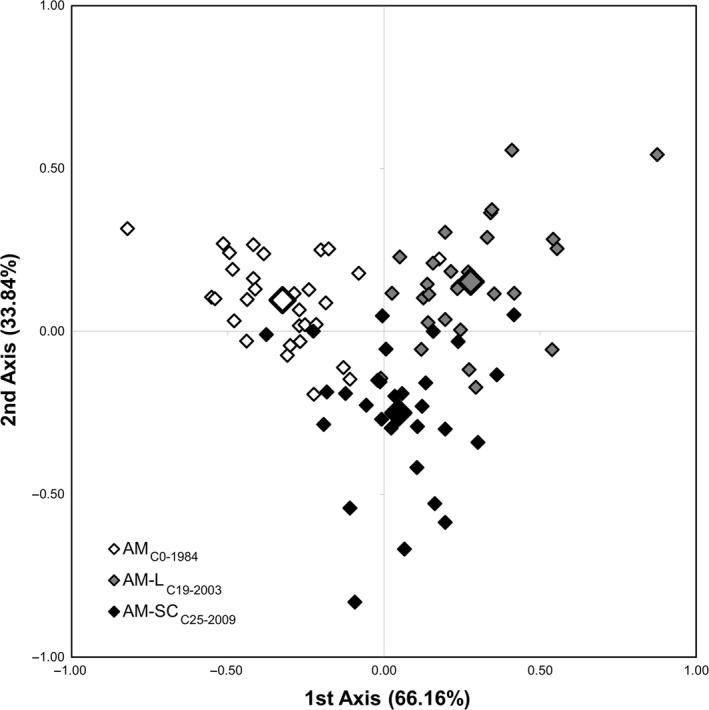
Factorial correspondence analysis (FCA) of 90 maize plants belonging to the Amiúdo cycles (initial population—AM_C__0‐1984_; AM‐L_C__19‐2003_ selection cycle; and AM‐SC_C__25‐2009_ selection cycle). Each individual genotype is indicated by a small symbol, while the cycle's mean value is represented by larger ones

**Figure 4:**
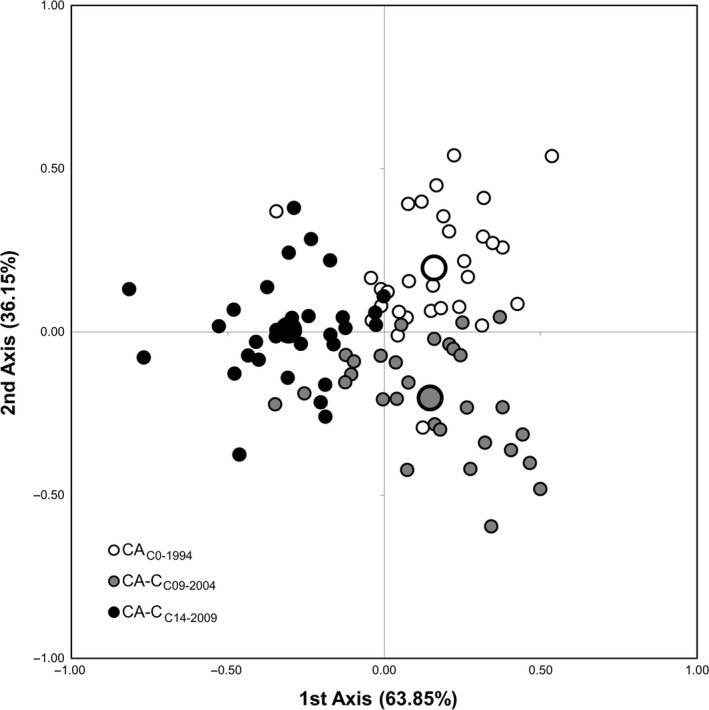
Factorial correspondence analysis (FCA) of 90 maize plants belonging to the Castro Verde cycles (initial population—CA_C__0‐1994_; CA‐C_C__09‐2004_ selection cycle; and CA‐C_C__14‐2009_ selection cycle). Each individual genotype is indicated by a small symbol, while the cycle's mean value is represented by larger ones

The factorial correspondence analysis depicts graphically the genetic proximity/differentiation within and among initial populations and selection cycles. From the factorial correspondence analysis of the Amiúdo population, the first axis, which accounted for 66.16% of the observed genotypic variance, separated the initial population (AM_C0‐1984_) from its selection cycles. The second axis, which accounted for 33.84% of the observed genotypic variance, separated the selection cycle from the Lousada site (AM‐L_C19‐2003_) from the selection cycle from the Serra do Carvalho site (AM‐SC_C25‐2009_; Figure 3). From the factorial correspondence analysis of Castro Verde, the first axis, which accounted for 63.85% of the observed genotypic variance, separated the most recent selection cycle (CA‐C_C14‐2009)_ from the other two. The second axis, which accounted for 36.15% of the observed genotypic variance, separated the initial cycle (CA_C0‐1994_) from the 2004 selection cycle (CA‐C_C09‐2004_; Figure 4).

### 3.3 Quality evolution

In relation to Amiúdo quality evaluation, the breeding activities led, in the material developed both at Lousada (AM‐L_C19‐2003_ cycle) and at Serra do Carvalho (AM‐SC_C25‐2009_ cycle), to a slight increase in the total carotenoid content (TCC) and in the color red/green index (a*), accompanied by a decrease in the levels of γ‐tocopherol (GT), protein (PR), fiber (FI), total volatile aldehydes (AL), total free phenolic (PH) compounds, *p*‐coumaric acid (CU), and ferulic acid (FE) (Table S8).

In the case of Castro Verde quality evaluation, although the results showed first a reduction of the flour's yellowness (taken as color parameter b* values) from CA_C0‐1994_ to CA‐C_C09‐2004_ and afterward from CA‐C_C09‐2004_ to CA‐C_C14‐2009_ cycle, the b* value stopped decreasing. Moreover, it was observed an increase in the levels of (α‐, δ‐, and γ‐) tocopherols (AT, DT, GT), and *p*‐coumaric acid (CU), as well as a decrease in the levels of fiber (FI), protein (PR), and total free phenolic (PH) compounds along the selection cycles. Nevertheless, for Castro Verde the majority of the quality traits (10 of 18) variation was erratic along selection cycles.

**Figure 5:**
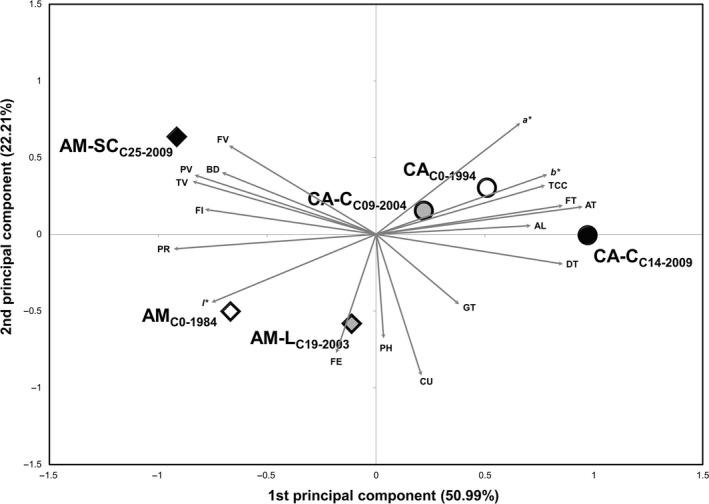
Biplot of principal component analysis (PCA) based on 18 quality traits in the Amiúdo cycles (initial population—AM_C__0‐1984_; AM‐L_C__19‐2003_ selection cycle; and AM‐SC_C__25‐2009_ selection cycle) and Castro Verde cycles (initial population—CA_C__0‐1994_; CA‐C_C__09‐2004_ selection cycle; and CA‐C_C__14‐2009_ selection cycle)

As for the principal component analysis based on the quality data in both the Amiúdo and Castro Verde populations, the first two components retained 73.20% of the total observed variance, with the first component explaining 50.99% of the observed variance (Figure 5). The traits that primarily influenced the first component were α‐ and δ‐tocopherol (AT and DT), fat (FT), peak and trough viscosities (PV and TV), and protein content (PR). The trait that primarily influenced the second component was the *p*‐coumaric acid (CU) content. The PCA biplot revealed an increase in the levels of α‐ and δ‐tocopherol (AT and DT) and fat (FT) when comparing the Amiúdo initial population (AM_C0‐1984_) with the Amiúdo cycle from the Lousada selection site (AM‐L_C19‐2003_). While comparing the Amiúdo initial population (AM_C0‐1984_) with the Amiúdo cycle from the Serra do Carvalho selection site (AM‐L_C25‐2009_), an opposite trend was depicted with a decrease in the levels of α‐ and δ‐tocopherol (AT and DT), and fat (FT), accompanied by a decrease in levels of *p*‐coumaric acid (CU).

## 4 DISCUSSION

Amiúdo and Castro Verde are two historical open‐pollinated maize populations that have been subjected to on‐farm stratified mass selection, in the context of a long‐term participatory breeding program. The results presented here revealed that this participatory program is improving or maintaining yield and quality parameters, while preserving genetic diversity of maize populations. Additionally, this program is empowering farmers as they keep the decision power and learn some basic population improvement methodologies, and at the same time represents an alternative strategy for endangered genetic resources’ on‐farm conservation.

### 4.1 Phenotypic effects of stratified mass selection

The results obtained from multilocation field trials, established to evaluate the effects of stratified mass selection in these two maize populations, showed that this methodology was able to improve the Amiúdo population, according to the established selection criteria in two different selection sites (Lousada and Serra do Carvalho). Nevertheless, according to the data collected, the same methodology failed to lead to an agronomic improvement of the Castro Verde population.

The Amiúdo population, integrated on the PPB program since its beginning, was selected by two different people, in two different selection sites, but with similar edaphic–climatic conditions. For both selection sites, achieving a higher‐yielding population was the breeding objective established by the farmer. Indeed, Amiúdo population had a yield increase through mass selection (0.8% gain per cycle) accompanied by heavier cobs and ears. This gain was however inferior to the experimental values obtained across long‐term maize recurrent selection methods for population improvement, as reviewed by Betrán, Bänziger, and Menz (2004). According to Betrán et al. (2004), when grain yield is the primary selection criterion, mass selection showed on average a 1.8% gain per cycle, being this value often smaller than the average values obtained with family‐based recurrent selection, such as selfed—S1 or S2—family selection (with 7% and 5% gain per cycle, respectively). One of the reasons for the slower yield progress observed in Amiúdo population in comparison with these reviewed values, besides its particular genetic background, may be a reflection of the lower selection intensity applied under the present participatory program (1%–5%).

As for Castro Verde population, the phenotypic data showed that stratified mass selection was able to partially induce phenotypic differences that follow the direction of the breeding objectives (maintenance of orange grain color set as breeding criterion after 2001). Nevertheless, an analysis of most of the other breeding criteria—achieve bigger ears, decrease the height of the ear insertion in the plants, and increase stalk resistance—showed that no significant improvements were obtained for the Castro Verde population using this methodology.

### 4.2 Implications for a quality‐oriented breeding program

An important aspect of both the Amiúdo and Castro Verde populations is the fact that their flours can be used for food. In fact, a recent sensory hedonic analysis of maize bread, including bread obtained from these populations, showed that both populations were able to produce bread with preferential characteristics (Carbas et al., 2016). With the objective of integrating these two populations in a quality‐oriented breeding program in due course, several traits related to consumer preferences and technological, nutritional, and organoleptic properties (quality traits) were measured. It was observed that the majority of those traits progressed erratically along the breeding program for the Castro Verde population. One exception was the total carotenoid content, which can be selected efficiently by choosing the more yellow/orange ears as the b* parameter (yellowness) is highly correlated with total carotenoid content (Kljaka, Grbešaa, & Karolyib, 2014). In general for quality traits, as the ones considered in this work, a direct visual selection, like the one performed for the agronomic traits, is not possible, and other complementary breeding methodologies are needed to encourage their effective improvement by farmers.

### 4.3 Breeding program weaknesses and strengths analysis

When grain yield was the primary breeding objective, on‐farm stratified mass selection, as described in this work, was effective in improving population yield although at a slower rate than what can be obtained through other more complex family‐based recurrent selection methods. With more diverse breeding objectives, as in the case of Castro Verde population, stratified mass selection was not always effective in achieving the same progress.

An extensive compilation of several cases of yield improvement achieved through mass selection in maize can be found at Hallauer et al. (2010, table 7.8, therein). A few examples that show the potential of stratified mass selection specifically in the context of a participatory maize breeding program were described in Mendes‐Moreira et al. (2008, 2009) and Smith et al. (2001). In the first two works, two other maize populations from the same Portuguese breeding program as in the present study had their agronomic performance improved in line with the farmers’ breeding objectives (Mendes‐Moreira et al., 2008, 2009). Also Smith et al. (2001) showed that tree cycles of stratified mass selection applied to five different Mexican maize populations were sufficient to obtain an increase in yield. Several factors have been identified as having an impact on mass selection effectiveness or ineffectiveness (Hallauer et al., 2010). Among them, one can highlight the trait under selection, an adequate isolation, the sample size utilized, genotype x environment interaction, and the precision of the experimental techniques used (environmental control, parental control). In the present work, it was shown that the selection methodology was able to alter traits related to ear architecture in the Amiúdo population, and therefore, the lack of agronomic progress in ear architecture‐related traits in the Castro Verde population should not be due to the trait under selection *per se*. Moreover, as the analysis of variance did not detect a significant genotype‐by‐environment interaction, the lack of Castro Verde progress should not be a consequence of this interaction. Instead, it could be most likely related to two particular aspects of the Castro Verde population: First, as the selection criterion until the year 2000 was set to get bigger ears, one hypothesis is that because this population had already ears of a significant size before entering the breeding program, the farmer was not fully engaged with the breeding activities. Second, after 2001, this population started to be selected at Coimbra site by the breeder. Therefore, another hypothesis for the lack of observable agronomic progress is that the population did not have adequate isolation, as other populations were also being grown at the same site; and the number of individual plants screened may have been too small to select/capture the best genotypes. Indeed, Castro Verde initial population, which resulted from years of farmers traditional selection based mainly on ear traits evaluated after harvest, had already a high grain yield for an open‐pollinated maize population (6,862.71 kg/ha). Probably due to this, a yield increase was not the main objective of the farmer involved on Castro Verde selection. This however was not the case for the farmer involved on Amiúdo selection that was aiming to improve the population initial yield (4,568.84 kg/ha). Nevertheless, both original maize populations showed on average higher yields than the only data publicly available on nonimproved historical Portuguese maize populations with high quality potential for maize bread *broa* production (Vaz Patto et al., 2007). Grain yield of these traditional populations was evaluated in a common‐garden field experiment, and it varied from 755 to 3,757 kg/ha, with an average of 1,982 kg/ha (Vaz Patto et al., 2007).

In the maize populations analyzed in the present study, not only natural selection but also human selection is affecting yield. In a review by Murphy, Carter, and Jones (2013), several examples of the effectiveness of evolutionary breeding (accounting only for natural selection) in improving the agronomic fitness of self‐pollinated cereal crops have been examined. With this breeding approach, improvement resulted from natural selection favoring high‐yielding genotypes as an outcome of the relationship between the yield capacity of an individual plant and its fitness components (Murphy et al., 2013). This yield increase is highly dependent on the selective environmental pressure and may affect maturity, plant height, and relationships among agronomic important traits unfavorably (Phillips & Wolfe, 2005). A comparison between the yield progress attained under the studied participatory breeding program and the yield progress that might be attained with an evolutionary breeding approach could have generated relevant information on the effectiveness of the human (artificial) selection versus natural selection. Unfortunately, no references were found in the literature on the effect of evolutionary breeding in maize populations to allow a direct comparison with the present study. However, by performing the selection of Amiúdo and Castro Verde populations within the target environment (at the farmers’ fields), on‐farm participatory breeding guarantees local adaptation and it may also counteract undesirable changes caused by natural selection in traits of agronomic importance. Moreover, by respecting farmers’ breeding objectives, an increase in the ratio of improved populations adopted by the farmer can be obtained.

Although one can argue that differences in response to selection in a similar genetic background may be due to different intensity or accuracy of selection, the acceptance and the enthusiasm of the farmers to join the program are the best guaranty of success. Farmers need to be fully engaged on the selection decision process (breeding objectives) but be open to accept breeder recommendations (preharvest parental control + postharvest selection).

One open question in the present study is: How able is the farmer to perform pre‐harvest trait selection? In the present work, the preharvest selection was not exclusive but mainly performed by the breeder, and therefore, the farmer's ability could not be clearly evaluated. Nevertheless, theoretically the preharvest selection methodologies proposed in the Portuguese participatory breeding program are very straightforward and are beforehand demonstrated by the breeder at the farmer's field. Therefore, these methodologies should be easily implemented by any farmer engaged in the breeding process. Indeed, it has been already demonstrated by Mendes‐Moreira et al. (2008) that such preharvest methodologies were successfully implemented by farmers in another maize population from the same participatory breeding program. The farmer's motivation and time availability/field dimensions (the bigger the field, the larger amount of time needed for stratified preharvest selection) seem to be the two main limitations for the successful implementation of this preharvest selection.

### 4.4 Genotypic effects of stratified mass selection

The effect of stratified mass selection in the genetic diversity levels of the two populations was also evaluated using SSRs. This analysis showed that the overall genetic diversity was maintained in both populations. In particular, even in the Amiúdo population where phenotypic modifications on ear morphology and yield gain were detected, no significant changes were identified on the overall genetic diversity levels, measured by the average number of alleles detected, observed and expected heterozygosity, and inbreeding coefficients. Also, no significant temporal variation of allele frequencies was detected in any of populations under study, indicating that the observed differences in allele frequency are more likely a result of genetic drift and/or sampling error (Waples, 1989a). As opposed to the results obtained by Labate et al. (1999) and Solomon et al. (2010), in which the authors detected a loss of genetic diversity in maize population subjected to few as 11 and 12 cycles of reciprocal recurrent selection, no significant differences in genetic diversity levels were identified in the current study. According to Hoban et al. (2014), changes in genetic diversity levels are most likely identified only when the effective population size is smaller than 100 individuals. In the present work, both populations had an effective population size bigger than 100, by contrast to the smaller effective population sizes estimated for the maize populations in Labate et al. (1999) and Solomon et al. (2010). In addition, the results presented here concur with the results previously described for the Portuguese Pigarro maize population (Vaz Patto et al., 2008) where stratified mass selection demonstrated to be an effective way to conserve diversity on‐farm, and at the same time allowed relevant phenotypic improvements to be achieved.

### 4.5 Final remarks

In conclusion, on‐farm stratified mass selection in the context of a participatory plant breeding program was shown to improve the agronomic performance of the Amiúdo population selected in two different selection sites. Moreover, for both the Amiúdo and Castro Verde populations, the breeding activities retained the populations’ genetic diversity. The unpredictability of the evolution of quality parameters along this breeding program also brings to light the need to develop efficient selection tools to maintain or improve these traits. Molecular markers associated with those traits and/or high throughput spectroscopy‐based phenotypic screening methodologies are among the tools that may aid in the improvement of characteristics that cannot be easily (visually) selected by farmers. The implementation of such breeding tools into participatory selection brings up another issue: To make these tools easily available, a platform of participatory research connecting enthusiastic, open‐minded farmers, breeders, and scientists must be built to make its application a reality.

## DATA ARCHIVING STATEMENT

Data available from the Dryad Digital Repository: https://doi.org/10.5061/dryad.nb320.

## References

[eva12549-bib-0001] AACC International (2012). Approved methods of analysis Method 14‐60.01, 11th ed. Total carotenoid content of cereal grains and flours. St. Paul, MN, USA: AACC International.

[eva12549-bib-0002] Belkhir, K. , Borsa, P. , Chikhi, L. , Raufaste, N. , & Bonhomme, F. (2004). GENETIX 4.05, logiciel sous Windows TM pour la génétique des populations. Laboratoire Génome, Populations, Interactions, CNRS UMR 5000, Université de Montpellier II, Montpellier (France).

[eva12549-bib-0003] Betrán, J. , Bänziger, M. , & Menz, M. (2004). Corn breeding In Wayne Smith C., Betrán J., & Runge E. C. A. (Eds.), Corn: Origin, history, technology, and production (pp. 305–398). New York, NY: Wiley.

[eva12549-bib-0004] Brites, C. , Trigo, M. J. , Santos, C. , Collar, C. , & Rosell, C. M. (2010). Maize‐based gluten‐free bread: Influence of processing parameters on sensory and instrumental quality. Food Bioprocess Technology, 3, 707–715.

[eva12549-bib-0005] Carbas, B. , Vaz Patto, M. C. , Bronze, M. R. , Bento da Silva, A. , Trigo, M. J. , & Brites, C. (2016). Maize flour parameters that are related to the consumer perceived quality of ‘broa’ specialty bread. Food Science and Technology, 36, 259–267.

[eva12549-bib-0006] Ceccarelli, S. (2015). Efficiency of plant breeding. Crop Science, 55, 87–97.

[eva12549-bib-0007] Ceccarelli, S. , Al‐Yassin, A. , Goldringer, I. , Mendes‐Moreira, P. , & Chable, V. (2012). Analysis of major PPB experiences worldwide. Retrieved from http://www.solibam.eu/modules/wfdownloads/singlefile.php?cid=12&lid=33

[eva12549-bib-0008] Ceccarelli, S. , Galie, A. , & Grando, S. (2013). Participatory breeding for climate change‐related traits In Kole C. (Ed.), Genomics and breeding for climate resilient crops. Concepts and strategies. Volume 1 (pp. 331–376). Heidelberg, Germany: Springer.

[eva12549-bib-0009] Ceccarelli, S. , Grando, S. , Maatougui, M. , Michae, L. M. , Slash, M. , Haghparast, R. , … Nachit, M. (2010). Plant breeding and climate changes. Journal of Agricultural Science, 148, 627–637.

[eva12549-bib-0010] Cleveland, D. A. , Soleri, D. , & Smith, S. E. (1999). Farmer plant breeding from biological perspective: Implications for collaborative plant breeding. CIMMYT Economics Working Paper CIMMYT, Mexico DF: CIMMYT, 99–10.

[eva12549-bib-0011] Do, C. , Waples, R. S. , Peel, D. , Macbeth, G. M. , Tillett, B. J. , & Ovenden, J. R. (2014). NeEstimator v2: Re‐implementation of software for the estimation of contemporary effective population size (Ne) from genetic data. Molecular Ecology Resources, 14, 209–214. 23992227 10.1111/1755-0998.12157

[eva12549-bib-0012] Excoffier, L. , Laval, G. , & Schneider, S. (2005). Arlequin ver. 3.0: An integrated software package for population genetics data analysis. Evolutionary Bioinformatics Online, 1, 47–50. PMC2658868 19325852

[eva12549-bib-0013] Excoffier, L. , Smouse, P. E. , & Quattro, J. M. (1992). Analysis of molecular variance inferred from metric distances among DNA haplotypes: Application to human mitochondrial DNA restriction sites. Genetics, 131, 479–491. 1644282 10.1093/genetics/131.2.479 PMC1205020

[eva12549-bib-0014] Fu, Y. B. (2015). Understanding crop genetic diversity under modern plant breeding. Theoretical and Applied Genetics, 128, 2131–2142. 26246331 10.1007/s00122-015-2585-y PMC4624815

[eva12549-bib-0015] Gardner, C. O. (1961). An evaluation of the effects of mass selection and seed irradiation with thermal neutrons on yield of corn. Crop Science, 1, 241–245.

[eva12549-bib-0016] Guo, S. W. , & Thompson, E. A. (1992). Performing the exact test of Hardy‐Weinberg proportion for multiple alleles. Biometrics, 48, 361–372. 1637966

[eva12549-bib-0017] Hallauer, A. R. (1999). Conversion of tropical germplasm for temperate area use. Illinois Corn Breeders School, 35, 20–36.

[eva12549-bib-0018] Hallauer, A. R. , Carena, M. J. , & Miranda Filho, J. D. (2010). Quantitative genetics in maize breeding (Vol. 6). New York, NY: Springer.

[eva12549-bib-0019] Hellin, J. , Bellon, M. R. , & Hearne, S. J. (2014). Maize landraces and adaptation to climate change in Mexico. Journal of Crop Improvement, 28, 484–501.

[eva12549-bib-0020] Hoban, S. , Arntzen, J. A. , Bruford, M. W. , Godoy, J. A. , Rus Hoelzel, A. , Segelbacher, G. , … Bertorelle, G. (2014). Comparative evaluation of potential indicators and temporal sampling protocols for monitoring genetic erosion. Evolutionary Applications, 7, 984–998. 25553062 10.1111/eva.12197 PMC4231590

[eva12549-bib-0021] ISO 9936 (2006). Animal and vegetable fats and oils – Determination of tocopherol and tocotrienol contents by high‐performance liquid chromatography.

[eva12549-bib-0022] Jarvis, D. I. , Hodgkin, T. , Sthapit, B. R. , Fadda, C. , & Lopez‐Noriega, I. (2011). An heuristic framework for identifying multiple ways of supporting the conservation and use of traditional crop varieties within the agricultural production system. Critical Reviews in Plant Sciences, 30, 125–176.

[eva12549-bib-0023] Khoury, C. K. , Bjorkman, A. D. , Dempewolf, H. , Ramirez‐Villegas, J. , Guarino, L. , Jarvis, A. , … Struik, P. C. (2014). Increasing homogeneity in global food supplies and the implications for food security. Proceedings of the National Academy of Sciences of the United States of America, 111, 4001–4006. 24591623 10.1073/pnas.1313490111 PMC3964121

[eva12549-bib-0501] Klensporf, D. , & Jelén, H. H. (2005). Analysis of volatile aldehydes in oat flakes by SPME‐GC/MS. Polish Journal of Food and Nutrition Sciences, 14, 389–395.

[eva12549-bib-0024] Kljaka, K. , Grbešaa, D. , & Karolyib, D. (2014). Reflectance colorimetry as a simple method for estimating carotenoid content in maize grain. Journal of Cereal Science, 59, 109–111.

[eva12549-bib-0025] Labate, J. A. , Lamkey, K. R. , Lee, M. , & Woodman, W. L. (1999). Temporal changes in allele frequencies in two reciprocally selected maize populations. Theoretical and Applied Genetics, 99, 1166–1178.

[eva12549-bib-0026] Lançon, J. , Pichaut, J. P. , Djaboutou, M. , Lewicki‐Dhainaut, S. , Viot, C. , & Lacape, J. M. (2008). Use of molecular markers in participatory plant breeding: Assessing the genetic variability in cotton populations bred by farmers. Annals of Applied Biology, 152, 113–119.

[eva12549-bib-0027] Lawrence, C. J. , Harper, L. C. , Schaeffer, M. L. , Sen, T. Z. , Seigfried, T. E. , & Campbell, D. A. (2008). MaizeGDB: The maize model organism database for basic, translational, and applied research. International Journal of Plant Genomics, 2008, 496957. 18769488 10.1155/2008/496957 PMC2518694

[eva12549-bib-0028] Lipka, A. E. , Gore, M. A. , Magallanes‐Lundback, M. , Mesberg, A. , Lin, H. , Tiede, T. , … DellaPenna, D. (2013). Genome‐wide association study and pathway‐level analysis of tocochromanol levels in maize grain. G3: Genes|Genomes|Genetics, 3, 1287–1299. 23733887 10.1534/g3.113.006148 PMC3737168

[eva12549-bib-0029] Liu, R. H. (2003). Health benefits of fruit and vegetables are from additive and synergistic combinations of phytochemicals. American Journal of Clinical Nutrition, 78, 517S–520S. 12936943 10.1093/ajcn/78.3.517S

[eva12549-bib-0030] Lopez‐Martinez, L. X. , Oliart‐Ros, R. M. , Valerio‐Alfaro, G. , Lee, C. H. , Parkin, K. L. , & Garcia, H. S. (2009). Antioxidant activity, phenolic compounds and anthocyanins content of eighteen strains of Mexican maize. Food Science and Technology, 42, 1187–1192.

[eva12549-bib-0031] Mendes‐Moreira, P. M. R. , Pego, S. E. , Vaz Patto, C. , & Hallauer, A. R. (2008). Comparison of selection methods on ‘Pigarro’, a Portuguese improved maize population with fasciation expression. Euphytica, 163, 481–499.

[eva12549-bib-0032] Mendes‐Moreira, P. M. R. , Vaz Patto, M. C. , Mota, M. , Mendes‐Moreira, J. , Santos, J. P. N. , Santos, J. P. P. , … Pego, S. E. (2009). ‘Fandango’: Long term adaptation of exotic germplasm to a Portuguese on‐farm‐conservation and breeding project. Maydica, 54, 269–285.

[eva12549-bib-0033] Murphy, K. M. , Carter, A. H. , & Jones, S. S. (2013). Evolutionary breeding and climate change In Kole C. (Ed.), Genomics and breeding for climate resilient crops. Concepts and strategies. Volume 1 (pp. 377–389). Heidelberg, Germany: Springer.

[eva12549-bib-0034] Nei, M. , & Tajima, F. (1981). Genetic drift and estimation of effective population size. Genetics, 98, 625–640. 17249104 10.1093/genetics/98.3.625 PMC1214463

[eva12549-bib-0035] Ortiz, R. (2011). Agrobiodiversity management for climate change In Lenné J. M., & Wood D. (Eds.), Agrobiodiversity management for food security (pp. 189–211). Wallingford, Oxon, United Kingdom: CAB International.

[eva12549-bib-0036] Owens, B. F. , Lipka, A. E. , Magallanes‐Lundback, M. , Tiede, T. , Diepenbrock, C. H. , Kandianis, C. B. , … Rocheford, T. (2014). A foundation for provitamin A biofortification of maize: Genome‐wide association and genomic prediction models of carotenoid levels. Genetics, 198, 1699–1716. 25258377 10.1534/genetics.114.169979 PMC4256781

[eva12549-bib-0037] Phillips, S. L. , & Wolfe, M. S. (2005). Evolutionary plant breeding for low input systems. The Journal of Agricultural Science, 143, 245–254.

[eva12549-bib-0038] Pingali, P. L. (ed.). (2001). CIMMYT 1999–2000 World Maize Facts and Trends. Meeting World Maize Needs: Technological Opportunities and Priorities for the Public Sector Mexico. D.F.: CIMMYT.

[eva12549-bib-0039] Rauf, S. , Teixeira da Silva, J. A. , Khan, A. A. , & Naveed, A. (2010). Consequences of plant breeding on genetic diversity. International Journal of Plant Breeding, 4, 1–21.

[eva12549-bib-0040] Raymond, M. , & Rousset, F. (1995). GENEPOP (version 1.2): Population genetics software for exact tests and ecumenicism. Journal of Heredity, 86, 248–249.

[eva12549-bib-0041] Rether, B. , Delmas, G. , & Laouedj, A. (1993). Isolation of polysaccharide‐free DNA from plants. Plant Molecular Biology Reporter, 11, 333–337.

[eva12549-bib-0042] Rice, W. R. (1989). Analyzing tables of statistical tests. Evolution, 43, 223–225. 28568501 10.1111/j.1558-5646.1989.tb04220.x

[eva12549-bib-0043] Saghai‐Maroof, M. A. , Soliman, K. M. , Jorgensen, R. A. , & Allard, R. W. (1984). Ribosomal DNA spacer‐length polymorphisms in barley: Mendelian inheritance, chromosomal location, and population dynamics. Proceedings of the National Academy of Sciences of the United States of America, 81, 8014–8018. 6096873 10.1073/pnas.81.24.8014 PMC392284

[eva12549-bib-0044] SAS Institute Inc (2004). The SAS system for windows, V8. 02. Cary, NC: SAS Institute.

[eva12549-bib-0045] Schuelke, M. (2000). An economic method for the fluorescent labeling of PCR fragments. Nature Biotechnology, 18, 233–234. 10.1038/72708 10657137

[eva12549-bib-0046] Silva, S. , Bronze, M. R. , Figueira, M. E. , Siwy, J. , Mischak, H. , Combet, E. , & Mullen, W. (2015). Impact of a 6‐wk olive oil supplementation in healthy adults on urinary proteomic biomarkers of coronary artery disease, chronic kidney disease, and diabetes (types 1 and 2): A randomized, parallel, controlled, double‐blind study. The American Journal of Clinical Nutrition, 101, 44–54. 25527749 10.3945/ajcn.114.094219

[eva12549-bib-0047] Silva, S. , Gomes, L. , Leitão, F. , Coelho, A. V. , & Vilas Boas, L. (2006). Phenolic compounds and antioxidant activity of *Olea europaea* L. fruits and leaves. Food Science Technology International, 12, 385–396.

[eva12549-bib-0048] Singleton, V. L. , Orthofer, R. , & Lamuela‐Raventos, R. M. (1999). Analysis of total phenols and other oxidant substrates and antioxidants by means of Folin‐Ciocalteu reagent. Methods in Enzymology, 299, 157–178.

[eva12549-bib-0049] Smith, M. E. , Castillo, F. G. , & Gómez, F. (2001). Participatory plant breeding with maize in Mexico and Honduras. Euphytica, 122, 551–565.

[eva12549-bib-0050] Solomon, K. F. , Martin, I. , & Zeppa, A. (2010). Temporal genetic structure patterns in tropical maize populations under reciprocal recurrent selection. Euphytica, 176, 239–249.

[eva12549-bib-0051] Vaz Patto, M. C. , Mendes‐Moreira, P. M. , Alves, M. L. , Mecha, E. , Brites, C. , Bronze, R. , & Pego, S. (2013). Participatory plant quality breeding: An ancient art revisited by knowledge sharing. The Portuguese experience In Andersen S. B. (Ed.), Plant breeding from laboratories to fields (pp. 255–288). Rijeka: InTech.

[eva12549-bib-0052] Vaz Patto, M. C. , Moreira, P. M. , Almeida, N. , Satovic, Z. , & Pego, S. (2008). Genetic diversity evolution through participatory maize breeding in Portugal. Euphytica, 161, 283–291.

[eva12549-bib-0053] Vaz Patto, M. C. , Moreira, P. M. , Carvalho, V. , & Pego, S. (2007). Collecting maize (*Zea mays* L. convar. *mays*) with potential technological ability for bread making in Portugal. Genetic Resources and Crop Evolution, 54, 1555–1563.

[eva12549-bib-0054] Waples, R. S. (1989a). Temporal variation in allele frequencies: Test the right hypothesis. Evolution, 43, 1236–1251. 28564497 10.1111/j.1558-5646.1989.tb02571.x

[eva12549-bib-0055] Waples, R. S. (1989b). A generalized approach for estimating effective population size from temporal changes in allele frequency. Genetics, 95, 489–502. 10.1093/genetics/121.2.379 PMC1203625 2731727

[eva12549-bib-0056] Wheeler, T. , & von Braun, J. (2013). Climate change impacts on global food security. Science, 341, 508–513. 23908229 10.1126/science.1239402

