# Supplementary material for: Long‐term on‐farm participatory maize breeding by stratified mass selection retains molecular diversity while improving agronomic performance
Source: Evol Appl. 2017 Oct 14;11(2):254–70. doi: 10.1111/eva.12549 (PMC5775497; doi:10.1111/eva.12549)
Supplement: Supplementary file 1 [file EVA-11-254-s001.docx]

**Supplementary materials**

Material and methods - Technological and chemical evaluation

*Total free phenolic content*

Ethanolic extracts (EtOH:H2O 50:50, v/v) for assessing the total phenolic content (PH) of maize flour were prepared following the procedure described by Lopez-Martinez at al. (2009), with some modifications. Briefly, 2 g of maize flour was extracted with 20 mL of EtOH:H2O (50:50, v/v) for 15 minutes, using an Ultra Turrax T25 (Janke & Kunkel, IKA Labortechnik, Germany). Final extracts were filtered using a Whatman filter paper (type42: retention 2.5 μm, diameter 18.5 cm). Extracts were prepared in triplicate and preserved at -20ºC until analysis.

*Volatile aldehydes content*

To one gram of maize flour, 4.5 mL of Milli-Q water was added. The volatile fraction of the flour was extracted from this mixture by solid phase micro extraction using a 2cm- 50/30 μm DVB/Carboxen/PDMS fiber (SUPELCO) with an exposure time of 60 minutes, at 60ºC.

Volatile compounds were analyzed in a GCMS-QP2010 Plus Shimadzu equipment and separated in a Varian Factor Four column (30 m x 0.25 mm x 0.25 μm). The injector was at 250ºC and the column was at 35ºC for 5 minutes, followed by a gradual increase of 5ºC/min until a final temperature of 230ºC was reached. Injection was performed using a splitless mode. The interface and ion source on MS equipment were set at 250ºC. Mass spectra were produced at 70 eV in a range of 29 – 299, using a scanning velocity of 555 scans/s. The mobile phase corresponded to helium at a flow rate of 2.1 mL/ min. The equipment was coupled to an automatic sampler AOC-5000 (Shimadzu). GCMSsolution Release 2.53SU1 software was applied for data acquisition and treatment.

Identification of volatile compounds was performed by a comparison of the experimental mass spectra with the ones from the software’s spectra library (WILEY 229, NIST 27 and 147). A standard mixture of hydrocarbons C8-C20 (40 mg/L each, in hexane) was prepared to determine linear retention indexes – LRI (Kovats indexes) – in order to confirm identification. The values of LRI determined for each compound were compared with described LRI for the same type of column (El-Sayed 2014, http://www.pherobase.com).

Table S1. Location, soil and climate characterization of the field trials sites.

|  | |  |  | Soil conditions* | | | | Climatic conditions** - rainfall (mm) and average temperature (ºC) | | | | | | |
| --- | --- | --- | --- | --- | --- | --- | --- | --- | --- | --- | --- | --- | --- | --- |
| Location | Year | Geographic coordinates  Latitude / Longitude | Altitude (m) | Dominant soil group | Topsoil pH | Topsoil organic carbon (% weight) | Available water storage capacity (mm) | Accumulated rainfall (mm) | Temp May (ºC) | Temp June (ºC) | Temp July (ºC) | Temp August (ºC) | Temp September (ºC) | Temp October (ºC) |
| Lousada | 2010 | 41º14'7.8''N / 8º18'11.1''W | 198 | Humic cambisols | 5.3 | 2.72 | 50 | 322.0 | 16 | 19 | 24 | 23 | 21 | 15 |
| Travassos | 2010 | 41°25'21.53"N / 7°50'42.91"W | 636 | Humic cambisols | 5.3 | 2.72 | 50 | 321.0 | 14 | 19 | 24 | 23 | 20 | 14 |
| Covas do Monte | 2010 | 40°53'19.68"N / 8°6'0.63"W | 455 | Humic cambisols | 5.3 | 2.72 | 50 | 323.5 | 14 | 19 | 24 | 23 | 20 | 14 |
| S. Pedro do Sul | 2010 | 40º47'7.91''N / 8º1'35.17''W | 475 | Humic cambisols | 5.3 | 2.72 | 50 | 325.5 | 14 | 19 | 24 | 23 | 20 | 14 |
| Vouzela-1 | 2010 | 40°42'35.1"N / 8°08'01.6"W | 461 | Humic cambisols | 5.3 | 2.72 | 50 | 323.5 | 16 | 19 | 24 | 23 | 20 | 15 |
| Vouzela-2 | 2010 | 40º42'25.31''N / 8º08'30.25''W | 450 | Humic cambisols | 5.3 | 2.72 | 50 | 323.5 | 16 | 19 | 24 | 23 | 20 | 15 |
| Quinta da Conraria | 2010 | 40º10'35.66''N / 8º23'44.80''W | 40 | Dystric regosols | 5.1 | 1.39 | 150 | 284.5 | 16 | 21 | 24 | 25 | 22 | 17 |
| Montemor-o-Velho | 2010 | 40º10'4.82''N / 8º41'14.84''W | 3 | Eutric fluvisols | 7.2 | 0.86 | 150 | 199.5 | 16 | 19 | 22 | 23 | 21 | 17 |
| Valada do Ribatejo | 2010 | 39º6'2.30''N / 8º47'30.27''W | 5 | Eutric fluvisols | 7.2 | 0.86 | 150 | 184.5 | 18 | 21 | 24 | 25 | 23 | 17 |
| Coimbra | 2009 | 40°13'0.22"N / 8°26'47.69"W | 21 | Chromic cambisols | 7.1 | 0.65 | 15 | 192.0 | 18 | 21 | 21 | 22 | 22 | 19 |

** Data from FAO/IIASA/ISRIC/ISSCAS/JRC, 2012. Harmonized World Soil Database (version 1.2).*

*** Data from (IPMA, I. P.). - Instituto Português do Mar e da Atmosfera, I. P (https://www.ipma.pt/en/oclima/monitorizacao). Accumulated rainfall and average temperatures registered during maize growing season (period extended from May until October).*

Table S2. Repeat motifs, bin location in the chromosome and number of alleles for 20 SSR loci used in the different initial populations and derived selection cycles.

|  |  |  | Amiúdo | | | | Castro Verde | | | |
| --- | --- | --- | --- | --- | --- | --- | --- | --- | --- | --- |
| Locus | Repeat motif | Bin | No. of alleles | | | |  | | | |
|  |  |  | AM_C0-1984_ | AM-L_C19-2003_ | AM-SC_C25-2009_ | Total | CA_C0-1994_ | CA-C_C09-2004_ | CA-C_C14-2009_ | Total |
| nc007 | CCT | 5.01 | 5 | 4 | 4 | 5 | 4 | 5 | 5 | 6 |
| phi059 | ACC | 10.02 | 3 | 2 | 2 | 3 | 3 | 2 | 3 | 3 |
| phi065 | CACTT | 9.03 | 3 | 3 | 3 | 3 | 3 | 4 | 4 | 4 |
| phi084 | GAA | 10.03-10.04 | 2 | 2 | 2 | 2 | 2 | 2 | 2 | 2 |
| umc1065 | ACA | 2.06 | 6 | 10 | 11 | 12 | 6 | 6 | 9 | 9 |
| umc1134 | AGC | 7.03 | 3 | 4 | 4 | 4 | 4 | 5 | 4 | 5 |
| umc1139 | GAC | 8.01 | 2 | 1 | 2 | 2 | 2 | 2 | 2 | 2 |
| umc1267 | CGG | 9.03-9.04 | 4 | 4 | 4 | 4 | 4 | 4 | 3 | 4 |
| umc1329 | GCC | 4.06 | 2 | 2 | 3 | 3 | 4 | 4 | 2 | 4 |
| umc1425 | TCA | 3.04 | 3 | 3 | 3 | 3 | 3 | 4 | 3 | 5 |
| umc1431 | GCA | 1.09 | 3 | 3 | 3 | 3 | 3 | 3 | 3 | 3 |
| umc1689 | GCG | 1.05 | 3 | 3 | 3 | 3 | 3 | 3 | 3 | 3 |
| umc1690 | GCA | 3.07 | 2 | 2 | 3 | 3 | 3 | 3 | 3 | 3 |
| umc1777 | CTG | 8.05 | 5 | 5 | 5 | 5 | 5 | 4 | 4 | 5 |
| umc1787 | CGG | 7.02 | 2 | 2 | 3 | 3 | 2 | 2 | 2 | 2 |
| umc2030 | CGA | 2.04 | 4 | 4 | 4 | 4 | 4 | 3 | 3 | 4 |
| umc2059 | CAG | 6.08 | 6 | 5 | 6 | 6 | 5 | 4 | 5 | 5 |
| umc2196 | CCG | 6.01 | 4 | 4 | 3 | 4 | 3 | 3 | 5 | 5 |
| umc2216 | TC | 5.06 | 5 | 3 | 4 | 5 | 5 | 4 | 3 | 6 |
| umc2281 | GTCC | 4.03 | 7 | 8 | 7 | 9 | 6 | 5 | 8 | 8 |
| Average nº alleles | | | 3.7 | 3.7 | 3.95 | 4.3 | 3.7 | 3.6 | 3.8 | 4.4 |
| Nº alleles lost (from the initial population) | | |  | 8 | 6 |  |  | 10 | 10 |  |
| Nº new alleles (from the initial population) | | |  | 8 | 11 |  |  | 8 | 12 |  |
| Total nº alleles | | | 74 | 74 | 79 | 86 | 74 | 72 | 76 | 88 |

Table S3. Distribution of the alleles across the Amiúdo and Castro Verde initial populations and derived selection cycles.

| Alleles | Amiúdo | | Castro Verde | |
| --- | --- | --- | --- | --- |
|  | Nº Alleles | Average frequency (%) | Nº Alleles | Average frequency (%) |
| Private alleles | 8 | 9.30 | 12 | 13.64 |
| Common to two cycles | 15 | 17.44 | 18 | 20.45 |
| Common to all cycles | 63 | 73.26 | 58 | 65.91 |
| Total | 86 | 100.00 | 88 | 100.00 |

Table S4. Inbreeding coefficients for the Amiúdo and Castro Verde populations per locus and per cycle and significance of the deviations from Hardy-Weinberg equilibrium.

| Locus | Amiúdo | | | Castro Verde | | |
| --- | --- | --- | --- | --- | --- | --- |
|  | AM_C0-1984_ | AM-L_C19-2003_ | AM-SC_C25-2009_ | CA_C0-1994_ | CA-C_C09-2004_ | CA-C_C14-2009_ |
| nc007 | -0.041 | 0.277 | 0.442 | 0.106 | 0.146 | 0.244 |
| phi059 | 0.096 | 0.133 | -0.101 | -0.084 | -0.094 | -0.033 |
| phi065 | 0.040 | -0.004 | -0.051 | 0.156 | -0.074 | 0.177 |
| phi084 | -0.152 | 0.085 | 0.210 | -0.349 | -0.094 | 0.500 |
| umc1065 | -0.166 | -0.005 | 0.151 | 0.056 | 0.106 | -0.055 |
| umc1134 | 0.202 | 0.035 | -0.086 | 0.122 | -0.061 | 0.277 |
| umc1139 | N/A^a^ | N/A | 0.208 | 0.360 | N/A | 0.205 |
| umc1267 | 0.248 | -0.288 | 0.029 | -0.042 | -0.013 | -0.025 |
| umc1329 | -0.118 | 0.045 | -0.182 | 0.086 | 0.144 | -0.012 |
| umc1425 | -0.036 | -0.140 | -0.121 | -0.030 | 0.256 | -0.097 |
| umc1431 | -0.129 | 0.104 | -0.005 | -0.012 | -0.024 | 0.283 |
| umc1689 | -0.096 | -0.037 | -0.025 | -0.086 | -0.039 | -0.083 |
| umc1690 | 0.149 | -0.264 | -0.039 | 0.069 | -0.143 | -0.136 |
| umc1777 | -0.050 | -0.079 | -0.040 | -0.066 | 0.190 | 0.083 |
| umc1787 | -0.206 | 0.065 | -0.084 | 0.114 | 0.100 | -0.177 |
| umc2030 | -0.193 | 0.023 | -0.027 | 0.143 | 0.445 | -0.062 |
| umc2059 | -0.150 | 0.130 | 0.364 | -0.239 | -0.074 | 0.141 |
| umc2196 | -0.164 | -0.176 | -0.220 | 0.055 | 0.251 | 0.209 |
| umc2216 | 0.618*** | 0.251 | 0.386 | 0.041 | 0.070 | -0.109 |
| umc2281 | -0.018 | -0.013 | -0.136 | -0.021 | -0.095 | 0.143 |

*Significant deviations from Hardy-Weinberg equilibrium after sequential Bonferroni corrections: “***” corresponds to significance at the 0.1% nominal level, “**” significance at the 1%, “*” significance at the 5%; no marking depicts non-significant values*

*^a^ N/A - Monomorphic (in AM-L_C19-2003_) or virtually monomorphic locus (AM_C0-1984_ and CA-C_C09-2004_: 29 out of 30 plants are homozygous)*

Table S5. Effective population size (Ne) for the Amiúdo population selected in the Lousada site (AMC0-1984 & AM-LC19-2003) and in the Serra do Carvalho site (AMC0-1984 & AM-SCC25-2009) and for the Castro Verde population (CAC0-1994 & CA-CC14-2009) as estimated by a temporal-based method under sample plan II.

|  | AM_C0-1984_ & AM-L_C19-2003_ | AM_C0-1984_ & AM-SC_C25-2009_ | CA_C0-1994_ & CA-C_C14-2009_ |
| --- | --- | --- | --- |
| *f̂* | 0.113 | 0.085 | 0.077 |
| S | 29.8 | 29.7 | 29.9 |
| N_e_ | 119.6 | 243.7 | 161.7 |
| 95% C.I. * | (68.2, 206.2) | (132.1, 467.7) | (82.8, 342.4) |

*f̂ - Nei & Tajima (1981) estimator of the standard variance in allele frequency change weighted over loci. The lowest allele frequency used was 0.05; S – Harmonized mean sample size; Ne – estimated effective population size; 95% C.I. - parametric chi-squared 95% confidence intervals for the estimated effective population size*

Table S6. Loci names and estimated allele frequencies for the 86 alleles detected. Waples’ (1989a) test for temporal variation in the allele frequencies distribution for the Lousada site between cycles AM_C0-1984_ and AM-L_C19-2003_ (effective population size, Ne=119.6, after 19 selection cycles) and for the Serra do Carvalho site between cycles AM_C0-1984_ and AM-SC_C25-2009_ (effective population size, Ne=243.7, after 25 selection cycles).

| Locus | Allele | AM_C0-1984_ allele frequency | AM-L_C19-2003_ allele frequency | AM-SC_C25-2009_ allele frequency | N_t0_^*^ | N_t19_^*^ | N_t25_^*^ | Temporal variation in allele frequency between AM_C0-1984_ & AM-L_C19-2003_^**^ | | | | Temporal variation in allele frequency between AM_C0-1984_ & AM-SC_C25-2009_^**^ | | | |
| --- | --- | --- | --- | --- | --- | --- | --- | --- | --- | --- | --- | --- | --- | --- | --- |
|  |  |  |  |  |  |  |  | (x-y)^2^ | var(x-y) | Χ^2^ | P-value | (x-y)^2^ | var(x-y) | Χ^2^ | P-value |
| nc007 | 1 | 0.18 | 0.33 | 0.20 | 30 | 30 | 30 | 0.02 | 0.02 | 1.08 | ns | 0.00 | 0.01 | 0.02 | ns |
|  | 2 | 0.25 | 0.15 | 0.22 | 30 | 30 | 30 | 0.01 | 0.02 | 0.58 | ns | 0.00 | 0.01 | 0.08 | ns |
|  | 3 | 0.45 | 0.48 | 0.52 | 30 | 30 | 30 | 0.00 | 0.03 | 0.04 | ns | 0.00 | 0.02 | 0.22 | ns |
|  | 4 | 0.08 | 0.00 | 0.07 | 30 | 30 | 30 | 0.01 | 0.00 | 1.60 | ns | 0.00 | 0.01 | 0.05 | ns |
|  | 5 | 0.03 | 0.03 | 0.00 | 30 | 30 | 30 | 0.00 | 0.00 | 0.00 | ns | 0.00 | 0.00 | 0.82 | ns |
| phi059 | 1 | 0.52 | 0.78 | 0.77 | 30 | 30 | 30 | 0.07 | 0.02 | 2.88 | ns | 0.06 | 0.02 | 3.29 | ns |
|  | 2 | 0.43 | 0.22 | 0.23 | 30 | 30 | 30 | 0.05 | 0.02 | 1.97 | ns | 0.04 | 0.02 | 2.18 | ns |
|  | 3 | 0.05 | 0.00 | 0.00 | 30 | 30 | 30 | 0.00 | 0.00 | 0.94 | ns | 0.00 | 0.00 | 1.24 | ns |
| phi065 | 1 | 0.38 | 0.52 | 0.58 | 30 | 30 | 30 | 0.02 | 0.03 | 0.66 | ns | 0.04 | 0.02 | 1.94 | ns |
|  | 2 | 0.03 | 0.07 | 0.02 | 30 | 30 | 30 | 0.00 | 0.01 | 0.22 | ns | 0.00 | 0.00 | 0.14 | ns |
|  | 3 | 0.58 | 0.42 | 0.40 | 30 | 30 | 30 | 0.03 | 0.03 | 1.02 | ns | 0.03 | 0.02 | 1.63 | ns |
| phi084 | 1 | 0.26 | 0.23 | 0.35 | 29 | 30 | 30 | 0.00 | 0.02 | 0.03 | ns | 0.01 | 0.02 | 0.47 | ns |
|  | 2 | 0.74 | 0.77 | 0.65 | 29 | 30 | 30 | 0.00 | 0.02 | 0.03 | ns | 0.01 | 0.02 | 0.47 | ns |
| umc1065 | 1 | 0.00 | 0.07 | 0.07 | 30 | 30 | 30 | 0.00 | 0.00 | 1.27 | ns | 0.00 | 0.00 | 1.67 | ns |
|  | 2 | 0.18 | 0.02 | 0.08 | 30 | 30 | 30 | 0.03 | 0.01 | 2.84 | ns | 0.01 | 0.01 | 1.05 | ns |
|  | 3 | 0.00 | 0.00 | 0.02 | 30 | 30 | 30 | − | − | − | − | 0.00 | 0.00 | 0.41 | ns |
|  | 4 | 0.00 | 0.03 | 0.02 | 30 | 30 | 30 | 0.00 | 0.00 | 0.62 | ns | 0.00 | 0.00 | 0.41 | ns |
|  | 5 | 0.00 | 0.05 | 0.00 | 30 | 30 | 30 | 0.00 | 0.00 | 0.94 | ns | − | − | − | − |
|  | 6 | 0.13 | 0.13 | 0.05 | 30 | 30 | 30 | 0.00 | 0.01 | 0.00 | ns | 0.01 | 0.01 | 1.01 | Ns |
|  | 7 | 0.38 | 0.45 | 0.57 | 30 | 30 | 30 | 0.00 | 0.03 | 0.17 | ns | 0.03 | 0.02 | 1.63 | Ns |
|  | 8 | 0.13 | 0.10 | 0.07 | 30 | 30 | 30 | 0.00 | 0.01 | 0.10 | ns | 0.00 | 0.01 | 0.60 | ns |
|  | 9 | 0.00 | 0.10 | 0.05 | 30 | 30 | 30 | 0.01 | 0.01 | 1.94 | ns | 0.00 | 0.00 | 1.24 | ns |
|  | 10 | 0.15 | 0.00 | 0.02 | 30 | 30 | 30 | 0.02 | 0.01 | 2.99 | ns | 0.02 | 0.01 | 2.82 | ns |
|  | 11 | 0.02 | 0.03 | 0.02 | 30 | 30 | 30 | 0.00 | 0.00 | 0.10 | ns | 0.00 | 0.00 | 0.00 | ns |
|  | 12 | 0.00 | 0.02 | 0.05 | 30 | 30 | 30 | 0.00 | 0.00 | 0.31 | ns | 0.00 | 0.00 | 1.24 | ns |
| umc1134 | 1 | 0.12 | 0.13 | 0.07 | 30 | 30 | 30 | 0.00 | 0.01 | 0.02 | ns | 0.00 | 0.01 | 0.36 | ns |
|  | 2 | 0.55 | 0.57 | 0.58 | 30 | 30 | 30 | 0.00 | 0.03 | 0.01 | ns | 0.00 | 0.02 | 0.05 | ns |
|  | 3 | 0.00 | 0.10 | 0.02 | 30 | 30 | 30 | 0.01 | 0.01 | 1.94 | ns | 0.00 | 0.00 | 0.41 | ns |
|  | 4 | 0.33 | 0.20 | 0.33 | 30 | 30 | 30 | 0.02 | 0.02 | 0.84 | ns | 0.00 | 0.02 | 0.00 | ns |
| umc1139 | 1 | 0.98 | 1.00 | 0.88 | 30 | 30 | 30 | 0.00 | 0.00 | 0.31 | ns | 0.01 | 0.01 | 1.95 | ns |
|  | 2 | 0.02 | 0.00 | 0.12 | 30 | 30 | 30 | 0.00 | 0.00 | 0.31 | ns | 0.01 | 0.01 | 1.95 | ns |
| umc1267 | 1 | 0.63 | 0.20 | 0.34 | 30 | 30 | 29 | 0.19 | 0.03 | 7.11 | ns | 0.08 | 0.02 | 4.01 | ns |
|  | 2 | 0.08 | 0.08 | 0.03 | 30 | 30 | 29 | 0.00 | 0.01 | 0.00 | ns | 0.00 | 0.00 | 0.51 | ns |
|  | 3 | 0.27 | 0.62 | 0.48 | 30 | 30 | 29 | 0.12 | 0.03 | 4.58 | ns | 0.05 | 0.02 | 2.40 | ns |
|  | 4 | 0.02 | 0.10 | 0.14 | 30 | 30 | 29 | 0.01 | 0.01 | 1.16 | ns | 0.01 | 0.01 | 2.51 | ns |
| umc1329 | 1 | 0.00 | 0.00 | 0.03 | 30 | 30 | 30 | − | − | − | − | 0.00 | 0.00 | 0.82 | ns |
|  | 2 | 0.52 | 0.60 | 0.75 | 30 | 30 | 30 | 0.01 | 0.03 | 0.26 | ns | 0.05 | 0.02 | 2.84 | ns |
|  | 3 | 0.48 | 0.40 | 0.22 | 30 | 30 | 30 | 0.01 | 0.03 | 0.26 | ns | 0.07 | 0.02 | 3.78 | ns |
| umc1425 | 1 | 0.03 | 0.13 | 0.18 | 30 | 30 | 30 | 0.01 | 0.01 | 1.21 | ns | 0.02 | 0.01 | 2.82 | ns |
|  | 2 | 0.03 | 0.02 | 0.03 | 30 | 30 | 30 | 0.00 | 0.00 | 0.10 | ns | 0.00 | 0.00 | 0.00 | ns |
|  | 3 | 0.93 | 0.85 | 0.78 | 30 | 30 | 30 | 0.01 | 0.01 | 0.66 | ns | 0.02 | 0.01 | 2.24 | ns |
| umc1431 | 1 | 0.50 | 0.57 | 0.66 | 30 | 30 | 29 | 0.00 | 0.03 | 0.16 | ns | 0.02 | 0.02 | 1.19 | ns |
|  | 2 | 0.33 | 0.20 | 0.22 | 30 | 30 | 29 | 0.02 | 0.02 | 0.84 | ns | 0.01 | 0.02 | 0.71 | ns |
|  | 3 | 0.17 | 0.23 | 0.12 | 30 | 30 | 29 | 0.00 | 0.02 | 0.26 | ns | 0.00 | 0.01 | 0.21 | ns |
| umc1689 | 1 | 0.22 | 0.33 | 0.28 | 30 | 30 | 30 | 0.01 | 0.02 | 0.63 | ns | 0.00 | 0.02 | 0.29 | ns |
|  | 2 | 0.03 | 0.05 | 0.08 | 30 | 30 | 30 | 0.00 | 0.00 | 0.06 | ns | 0.00 | 0.00 | 0.55 | ns |
|  | 3 | 0.75 | 0.62 | 0.63 | 30 | 30 | 30 | 0.02 | 0.02 | 0.76 | ns | 0.01 | 0.02 | 0.77 | ns |
| umc1690 | 1 | 0.52 | 0.45 | 0.42 | 30 | 30 | 30 | 0.00 | 0.03 | 0.16 | ns | 0.01 | 0.02 | 0.49 | ns |
|  | 2 | 0.00 | 0.00 | 0.02 | 30 | 30 | 30 | − | − | − | − | 0.00 | 0.00 | 0.41 | ns |
|  | 3 | 0.48 | 0.55 | 0.57 | 30 | 30 | 30 | 0.00 | 0.03 | 0.16 | ns | 0.01 | 0.02 | 0.34 | ns |
| umc1777 | 1 | 0.07 | 0.05 | 0.10 | 30 | 30 | 30 | 0.00 | 0.01 | 0.05 | ns | 0.00 | 0.01 | 0.18 | ns |
|  | 2 | 0.58 | 0.67 | 0.58 | 30 | 30 | 30 | 0.01 | 0.03 | 0.27 | ns | 0.00 | 0.02 | 0.00 | ns |
|  | 3 | 0.23 | 0.17 | 0.20 | 30 | 30 | 30 | 0.00 | 0.02 | 0.26 | ns | 0.00 | 0.01 | 0.08 | ns |
|  | 4 | 0.05 | 0.08 | 0.10 | 30 | 30 | 30 | 0.00 | 0.01 | 0.16 | ns | 0.00 | 0.01 | 0.44 | ns |
|  | 5 | 0.07 | 0.03 | 0.02 | 30 | 30 | 30 | 0.00 | 0.01 | 0.22 | ns | 0.00 | 0.00 | 0.76 | ns |
| umc1787 | 1 | 0.00 | 0.00 | 0.05 | 30 | 30 | 30 | − | − | − | − | 0.00 | 0.00 | 1.24 | ns |
|  | 2 | 0.57 | 0.35 | 0.35 | 30 | 30 | 30 | 0.05 | 0.03 | 1.74 | ns | 0.05 | 0.02 | 2.29 | ns |
|  | 3 | 0.43 | 0.65 | 0.60 | 30 | 30 | 30 | 0.05 | 0.03 | 1.74 | ns | 0.03 | 0.02 | 1.35 | ns |
| umc2030 | 1 | 0.02 | 0.12 | 0.05 | 30 | 29 | 30 | 0.01 | 0.01 | 1.57 | ns | 0.00 | 0.00 | 0.42 | ns |
|  | 2 | 0.18 | 0.21 | 0.10 | 30 | 29 | 30 | 0.00 | 0.02 | 0.03 | ns | 0.01 | 0.01 | 0.69 | ns |
|  | 3 | 0.58 | 0.62 | 0.75 | 30 | 29 | 30 | 0.00 | 0.03 | 0.05 | ns | 0.03 | 0.02 | 1.51 | ns |
|  | 4 | 0.22 | 0.05 | 0.10 | 30 | 29 | 30 | 0.03 | 0.01 | 2.13 | ns | 0.01 | 0.01 | 1.24 | ns |
| umc2059 | 1 | 0.18 | 0.07 | 0.23 | 30 | 30 | 30 | 0.01 | 0.01 | 1.14 | ns | 0.00 | 0.01 | 0.18 | ns |
|  | 2 | 0.05 | 0.17 | 0.05 | 30 | 30 | 30 | 0.01 | 0.01 | 1.30 | ns | 0.00 | 0.00 | 0.00 | ns |
|  | 3 | 0.20 | 0.15 | 0.15 | 30 | 30 | 30 | 0.00 | 0.02 | 0.16 | ns | 0.00 | 0.01 | 0.21 | ns |
|  | 4 | 0.25 | 0.27 | 0.25 | 30 | 30 | 30 | 0.00 | 0.02 | 0.01 | ns | 0.00 | 0.02 | 0.00 | ns |
|  | 5 | 0.23 | 0.35 | 0.30 | 30 | 30 | 30 | 0.01 | 0.02 | 0.61 | ns | 0.00 | 0.02 | 0.28 | ns |
|  | 6 | 0.08 | 0.00 | 0.02 | 30 | 30 | 30 | 0.01 | 0.00 | 1.60 | ns | 0.00 | 0.00 | 1.13 | ns |
| umc2196 | 1 | 0.05 | 0.02 | 0.00 | 30 | 30 | 30 | 0.00 | 0.00 | 0.32 | ns | 0.00 | 0.00 | 1.24 | ns |
|  | 2 | 0.60 | 0.43 | 0.58 | 30 | 30 | 30 | 0.03 | 0.03 | 1.02 | ns | 0.00 | 0.02 | 0.01 | ns |
|  | 3 | 0.10 | 0.47 | 0.15 | 30 | 30 | 30 | 0.13 | 0.02 | 6.10 | ns | 0.00 | 0.01 | 0.28 | ns |
|  | 4 | 0.25 | 0.08 | 0.27 | 30 | 30 | 30 | 0.03 | 0.02 | 1.84 | ns | 0.00 | 0.02 | 0.02 | ns |
| umc2216 | 1 | 0.52 | 0.48 | 0.69 | 28 | 29 | 26 | 0.00 | 0.03 | 0.04 | ns | 0.03 | 0.02 | 1.47 | ns |
|  | 2 | 0.25 | 0.29 | 0.13 | 28 | 29 | 26 | 0.00 | 0.02 | 0.09 | ns | 0.01 | 0.01 | 0.99 | ns |
|  | 3 | 0.04 | 0.22 | 0.08 | 28 | 29 | 26 | 0.04 | 0.01 | 2.82 | ns | 0.00 | 0.00 | 0.38 | ns |
|  | 4 | 0.02 | 0.00 | 0.00 | 28 | 29 | 26 | 0.00 | 0.00 | 0.33 | ns | 0.00 | 0.00 | 0.40 | ns |
|  | 5 | 0.18 | 0.00 | 0.10 | 28 | 29 | 26 | 0.03 | 0.01 | 3.61 | ns | 0.01 | 0.01 | 0.66 | ns |
| umc2281 | 1 | 0.05 | 0.25 | 0.28 | 29 | 30 | 30 | 0.04 | 0.01 | 2.79 | ns | 0.05 | 0.01 | 4.58 | ns |
|  | 2 | 0.02 | 0.13 | 0.03 | 29 | 30 | 30 | 0.01 | 0.01 | 1.75 | ns | 0.00 | 0.00 | 0.13 | ns |
|  | 3 | 0.07 | 0.02 | 0.02 | 29 | 30 | 30 | 0.00 | 0.00 | 0.62 | ns | 0.00 | 0.00 | 0.81 | ns |
|  | 4 | 0.00 | 0.05 | 0.02 | 29 | 30 | 30 | 0.00 | 0.00 | 0.92 | ns | 0.00 | 0.00 | 0.40 | ns |
|  | 5 | 0.03 | 0.03 | 0.00 | 29 | 30 | 30 | 0.00 | 0.00 | 0.00 | ns | 0.00 | 0.00 | 0.86 | ns |
|  | 6 | 0.33 | 0.20 | 0.35 | 29 | 30 | 30 | 0.02 | 0.02 | 0.77 | ns | 0.00 | 0.02 | 0.03 | ns |
|  | 7 | 0.47 | 0.25 | 0.27 | 29 | 30 | 30 | 0.05 | 0.03 | 1.86 | ns | 0.04 | 0.02 | 2.05 | ns |
|  | 8 | 0.00 | 0.07 | 0.03 | 29 | 30 | 30 | 0.00 | 0.00 | 1.24 | ns | 0.00 | 0.00 | 0.80 | ns |
|  | 9 | 0.03 | 0.00 | 0.00 | 29 | 30 | 30 | 0.00 | 0.00 | 0.65 | ns | 0.00 | 0.00 | 0.86 | ns |

** N_t0_ equals sample size at AM_C0-1984_, N_t19_ equals sample size at AM-L_C19-2003_ and N_t25_ equals sample size at AM-SC_C25-2009_*

*** The test statistic follows a Χ^2^ distribution and is calculated as Χ^2^=(x-y) ^2^/var(x-y), where x is the estimated allele frequency in the initial sample (AM_C0-1984_), y is the estimated allele frequency in the following sample (AM-L_C19-2003_, and AM-SC_C25-2009_), and var(x-y) is the variance of the difference in the frequencies between the two time points. P-value obtained after sequential Bonferroni corrections: ns - non-significant; * - significant at P < 0.05; ** - significant at P < 0.01; *** - significant at P < 0.001*

Table S7. Loci names and estimated allele frequencies for the 86 alleles detected between CA_C0-1994_ and CA-C_C14-2009_ selection cycles. Waples’ (1989a) test for temporal variation in the allele frequencies distribution between cycles CA_C0-1994_ and CA-C_C14-2009_ (effective population size, Ne= 161.7, after 14 selection cycles).

| Locus | Allele | CA_C0-1994_ allele frequency | CA-C_C14-2009_ allele frequency | N_t0_^*^ | N_t14_^*^ | Temporal variation in allele frequency between CA_C0-1994_ & CA-C_C14-2009_^**^ | | | |
| --- | --- | --- | --- | --- | --- | --- | --- | --- | --- |
|  |  |  |  |  |  | (x-y)^2^ | v(x-y) | Χ^2^ | P-value |
| nc007 | 1 | 0.133 | 0.017 | 30 | 30 | 0.014 | 0.005 | 2.611 | ns |
|  | 2 | 0.350 | 0.500 | 30 | 30 | 0.023 | 0.018 | 1.227 | ns |
|  | 3 | 0.383 | 0.233 | 30 | 30 | 0.023 | 0.016 | 1.406 | ns |
|  | 4 | 0.000 | 0.033 | 30 | 30 | 0.001 | 0.001 | 0.902 | ns |
|  | 5 | 0.133 | 0.217 | 30 | 30 | 0.007 | 0.011 | 0.642 | ns |
| phi059 | 1 | 0.600 | 0.567 | 30 | 30 | 0.001 | 0.018 | 0.061 | ns |
|  | 2 | 0.350 | 0.367 | 30 | 30 | 0.000 | 0.017 | 0.016 | ns |
|  | 3 | 0.050 | 0.067 | 30 | 30 | 0.000 | 0.004 | 0.068 | ns |
| phi065 | 1 | 0.917 | 0.867 | 30 | 30 | 0.002 | 0.007 | 0.345 | ns |
|  | 2 | 0.000 | 0.017 | 30 | 30 | 0.000 | 0.001 | 0.449 | ns |
|  | 3 | 0.017 | 0.017 | 30 | 30 | 0.000 | 0.001 | 0.000 | ns |
|  | 4 | 0.067 | 0.100 | 30 | 30 | 0.001 | 0.006 | 0.193 | ns |
| phi084 | 1 | 0.267 | 0.350 | 30 | 30 | 0.007 | 0.016 | 0.434 | ns |
|  | 2 | 0.733 | 0.650 | 30 | 30 | 0.007 | 0.016 | 0.434 | ns |
| umc1065 | 1 | 0.017 | 0.033 | 30 | 30 | 0.000 | 0.002 | 0.151 | ns |
|  | 2 | 0.000 | 0.083 | 30 | 30 | 0.007 | 0.003 | 2.316 | ns |
|  | 4 | 0.133 | 0.117 | 30 | 30 | 0.000 | 0.008 | 0.034 | ns |
|  | 5 | 0.017 | 0.017 | 30 | 30 | 0.000 | 0.001 | 0.000 | ns |
|  | 6 | 0.517 | 0.417 | 30 | 30 | 0.010 | 0.019 | 0.535 | ns |
|  | 7 | 0.017 | 0.033 | 30 | 30 | 0.000 | 0.002 | 0.151 | ns |
|  | 8 | 0.300 | 0.233 | 30 | 30 | 0.004 | 0.015 | 0.303 | ns |
|  | 9 | 0.000 | 0.033 | 30 | 30 | 0.001 | 0.001 | 0.902 | ns |
|  | 10 | 0.000 | 0.033 | 30 | 30 | 0.001 | 0.001 | 0.902 | ns |
| umc1134 | 1 | 0.000 | 0.050 | 30 | 30 | 0.003 | 0.002 | 1.367 | ns |
|  | 2 | 0.017 | 0.000 | 30 | 30 | 0.000 | 0.001 | 0.449 | ns |
|  | 3 | 0.750 | 0.617 | 30 | 30 | 0.018 | 0.016 | 1.094 | ns |
|  | 4 | 0.117 | 0.267 | 30 | 30 | 0.023 | 0.012 | 1.935 | ns |
|  | 5 | 0.117 | 0.067 | 30 | 30 | 0.003 | 0.006 | 0.400 | ns |
| umc1139 | 1 | 0.917 | 0.879 | 30 | 29 | 0.001 | 0.007 | 0.203 | ns |
|  | 2 | 0.083 | 0.121 | 30 | 29 | 0.001 | 0.007 | 0.203 | ns |
| umc1267 | 1 | 0.450 | 0.400 | 30 | 30 | 0.003 | 0.018 | 0.136 | ns |
|  | 2 | 0.050 | 0.200 | 30 | 30 | 0.023 | 0.008 | 2.741 | ns |
|  | 3 | 0.317 | 0.400 | 30 | 30 | 0.007 | 0.017 | 0.402 | ns |
|  | 4 | 0.183 | 0.000 | 30 | 30 | 0.034 | 0.006 | 5.378 | ns |
| umc1329 | 1 | 0.017 | 0.000 | 29 | 30 | 0.000 | 0.001 | 0.467 | ns |
|  | 2 | 0.397 | 0.417 | 29 | 30 | 0.000 | 0.018 | 0.022 | ns |
|  | 3 | 0.569 | 0.583 | 29 | 30 | 0.000 | 0.018 | 0.011 | ns |
|  | 4 | 0.017 | 0.000 | 29 | 30 | 0.000 | 0.001 | 0.467 | ns |
| umc1425 | 1 | 0.117 | 0.050 | 30 | 30 | 0.004 | 0.006 | 0.776 | ns |
|  | 2 | 0.233 | 0.000 | 30 | 30 | 0.054 | 0.008 | 7.038 | ns |
|  | 4 | 0.650 | 0.867 | 30 | 30 | 0.047 | 0.014 | 3.414 | ns |
|  | 5 | 0.000 | 0.083 | 30 | 30 | 0.007 | 0.003 | 2.316 | ns |
| umc1431 | 1 | 0.672 | 0.667 | 29 | 30 | 0.000 | 0.017 | 0.002 | ns |
|  | 2 | 0.052 | 0.017 | 29 | 30 | 0.001 | 0.002 | 0.495 | ns |
|  | 3 | 0.276 | 0.317 | 29 | 30 | 0.002 | 0.016 | 0.106 | ns |
| umc1689 | 1 | 0.467 | 0.417 | 30 | 30 | 0.003 | 0.019 | 0.135 | ns |
|  | 2 | 0.217 | 0.133 | 30 | 30 | 0.007 | 0.011 | 0.642 | ns |
|  | 3 | 0.317 | 0.450 | 30 | 30 | 0.018 | 0.018 | 1.002 | ns |
|  | 1 | 0.183 | 0.150 | 30 | 30 | 0.001 | 0.010 | 0.106 | ns |
|  | 2 | 0.033 | 0.100 | 30 | 30 | 0.004 | 0.005 | 0.953 | ns |
|  | 3 | 0.783 | 0.750 | 30 | 30 | 0.001 | 0.013 | 0.083 | ns |
| umc1777 | 1 | 0.217 | 0.167 | 30 | 30 | 0.003 | 0.012 | 0.215 | ns |
|  | 2 | 0.650 | 0.600 | 30 | 30 | 0.003 | 0.018 | 0.142 | ns |
|  | 3 | 0.067 | 0.200 | 30 | 30 | 0.018 | 0.009 | 2.049 | ns |
|  | 4 | 0.017 | 0.000 | 30 | 30 | 0.000 | 0.001 | 0.449 | ns |
|  | 5 | 0.050 | 0.033 | 30 | 30 | 0.000 | 0.003 | 0.093 | ns |
| umc1787 | 1 | 0.717 | 0.733 | 30 | 30 | 0.000 | 0.015 | 0.018 | ns |
|  | 2 | 0.283 | 0.267 | 30 | 30 | 0.000 | 0.015 | 0.018 | ns |
| umc2030 | 1 | 0.050 | 0.100 | 30 | 30 | 0.003 | 0.005 | 0.480 | ns |
|  | 2 | 0.733 | 0.800 | 30 | 30 | 0.004 | 0.013 | 0.331 | ns |
|  | 3 | 0.200 | 0.100 | 30 | 30 | 0.010 | 0.010 | 1.045 | ns |
|  | 4 | 0.017 | 0.000 | 30 | 30 | 0.000 | 0.001 | 0.449 | ns |
| umc2059 | 1 | 0.650 | 0.450 | 30 | 30 | 0.040 | 0.019 | 2.154 | ns |
|  | 2 | 0.183 | 0.100 | 30 | 30 | 0.007 | 0.009 | 0.760 | ns |
|  | 3 | 0.050 | 0.100 | 30 | 30 | 0.003 | 0.005 | 0.480 | ns |
|  | 4 | 0.017 | 0.050 | 30 | 30 | 0.001 | 0.002 | 0.458 | ns |
|  | 5 | 0.100 | 0.300 | 30 | 30 | 0.040 | 0.012 | 3.331 | ns |
| umc2196 | 1 | 0.000 | 0.017 | 30 | 29 | 0.000 | 0.001 | 0.467 | ns |
|  | 2 | 0.050 | 0.069 | 30 | 29 | 0.000 | 0.004 | 0.086 | ns |
|  | 3 | 0.300 | 0.586 | 30 | 29 | 0.082 | 0.019 | 4.396 | ns |
|  | 4 | 0.000 | 0.017 | 30 | 29 | 0.000 | 0.001 | 0.467 | ns |
|  | 5 | 0.650 | 0.310 | 30 | 29 | 0.115 | 0.019 | 6.113 | ns |
| umc2216 | 1 | 0.717 | 0.862 | 30 | 29 | 0.021 | 0.013 | 1.675 | ns |
|  | 2 | 0.000 | 0.035 | 30 | 29 | 0.001 | 0.001 | 0.944 | ns |
|  | 3 | 0.200 | 0.103 | 30 | 29 | 0.009 | 0.010 | 0.955 | ns |
|  | 4 | 0.033 | 0.000 | 30 | 29 | 0.001 | 0.001 | 0.881 | ns |
|  | 5 | 0.017 | 0.000 | 30 | 29 | 0.000 | 0.001 | 0.438 | ns |
|  | 6 | 0.033 | 0.000 | 30 | 29 | 0.001 | 0.001 | 0.881 | ns |
| umc2281 | 1 | 0.167 | 0.233 | 30 | 30 | 0.004 | 0.012 | 0.369 | ns |
|  | 2 | 0.067 | 0.017 | 30 | 30 | 0.003 | 0.003 | 0.834 | ns |
|  | 3 | 0.617 | 0.383 | 30 | 30 | 0.054 | 0.019 | 2.904 | ns |
|  | 4 | 0.000 | 0.017 | 30 | 30 | 0.000 | 0.001 | 0.449 | ns |
|  | 5 | 0.067 | 0.100 | 30 | 30 | 0.001 | 0.006 | 0.193 | ns |
|  | 6 | 0.017 | 0.100 | 30 | 30 | 0.007 | 0.004 | 1.683 | ns |
|  | 7 | 0.067 | 0.117 | 30 | 30 | 0.003 | 0.006 | 0.400 | ns |
|  | 8 | 0.000 | 0.033 | 30 | 30 | 0.001 | 0.001 | 0.902 | ns |

** N_t0_ equals sample size at CA_C0-1994_, and N_t14_ equals sample size at CA-C_C14-2009_*

*** The test statistic follows a Χ^2^ distribution and is calculated as Χ^2^=(x-y) ^2^/var(x-y), where x is the estimated allele frequency in the initial sample (CA_C0-1994_), y is the estimated allele frequency in the following sample (CA-C_C14-2009_), and var(x-y) is the variance of the difference in the frequencies between the two time points. P-value obtained after sequential Bonferroni corrections: ns - non-significant; * - significant at P < 0.05; ** - significant at P < 0.01; *** - significant at P < 0.001*

Table S8. Quality traits mean values for the Amiúdo and Castro Verde initial population (AMC0-1984 and CAC0-1994, respectively) and derived selection cycles.

|  | Amiúdo | | | Castro Verde | | |
| --- | --- | --- | --- | --- | --- | --- |
| Quality trait | AM_C0-1984_ | AM-L_C19-2003_ | AM-SC_C25-2009_ | CA_C0-1994_ | CA-C_C09-2004_ | CA-C_C14-2009_ |
| red/green index (*a**) | -1.12 | -0.97 | -0.30 | 0.49 | -0.03 | 0.12 |
| yellow/blue index (*b**) | 31.38 | 30.85 | 31.15 | 35.92 | 33.62 | 34.08 |
| Lightness (*L**) | 84.70 | 85.38 | 84.43 | 83.06 | 84.24 | 82.51 |
| α-tocopherol (AT) | 22.75 | 27.74 | 11.64 | 82.17 | 84.22 | 106.57 |
| δ-tocopherol (DT) | 18.04 | 18.69 | 13.87 | 18.67 | 22.62 | 28.1 |
| γ-tocopherol (GT) | 233.45 | 220.5 | 156.25 | 125.16 | 261.65 | 301.31 |
| Protein (PR) | 12.75 | 11.35 | 12.23 | 11.18 | 11.05 | 9.79 |
| Fiber (FI) | 2.39 | 2.19 | 2.36 | 2.31 | 2.25 | 2.00 |
| Fat (FT) | 4.83 | 4.97 | 4.83 | 5.16 | 5.16 | 5.08 |
| Total carotenoids (TCC) | 49.53 | 50.24 | 51.15 | 75.21 | 51.78 | 69.82 |
| Aldehydes (AL) | 1888416 | 601334 | 1296426 | 2642322 | 1609767 | 10584651 |
| Total free phenolics (PH) | 164.54 | 156.81 | 107.51 | 160.67 | 133.68 | 121.79 |
| *p*-coumaric acid (CU) | 0.32 | 0.29 | 0.11 | 0.17 | 0.19 | 0.29 |
| ferulic acid (FE) | 0.34 | 0.18 | 0.07 | 0.13 | 0.07 | 0.17 |
| Breakdown (BD) | 343 | 188 | 624 | 150 | 121 | 256 |
| Trough viscosity (TV) | 1564 | 1029 | 1746 | 1073 | 1340 | 1036 |
| Peak viscosity (PV) | 1907 | 1217 | 2370 | 1223 | 1461 | 1292 |
| Final viscosity (FV) | 4860 | 3190 | 6135 | 3747 | 4889 | 3792 |

*Quality traits’ units: Red/green index (a*) – if a* is positive it means that samples tend to the red part of the color spectra; Yellow/blue index (b*) – if b* is positive it means that samples tend to the yellow part of the color spectra; Lightness (L*) – varies from L* = 0 (black) to L* = 100 (white); Tocopherols (AT, DT and GT) expressed in μg/g fat basis; Protein (PR), fiber (FI) and fat (FT) expressed in percentage; Total carotenoids (TCC) expressed in μgrams of lutein equivalent per gram of sample; Aldehydes (AL) taken as the chromatogram peak area; Total free phenolics (PH) expressed in gallic acid equivalents/100 g of dry weight; p-coumaric acid (CU) and ferulic acid (FE) expressed in mg/100 g of dry weight; Viscosity parameters (BD, TV, PV and FV) expressed in cPoise*

Figures

Figure S1.


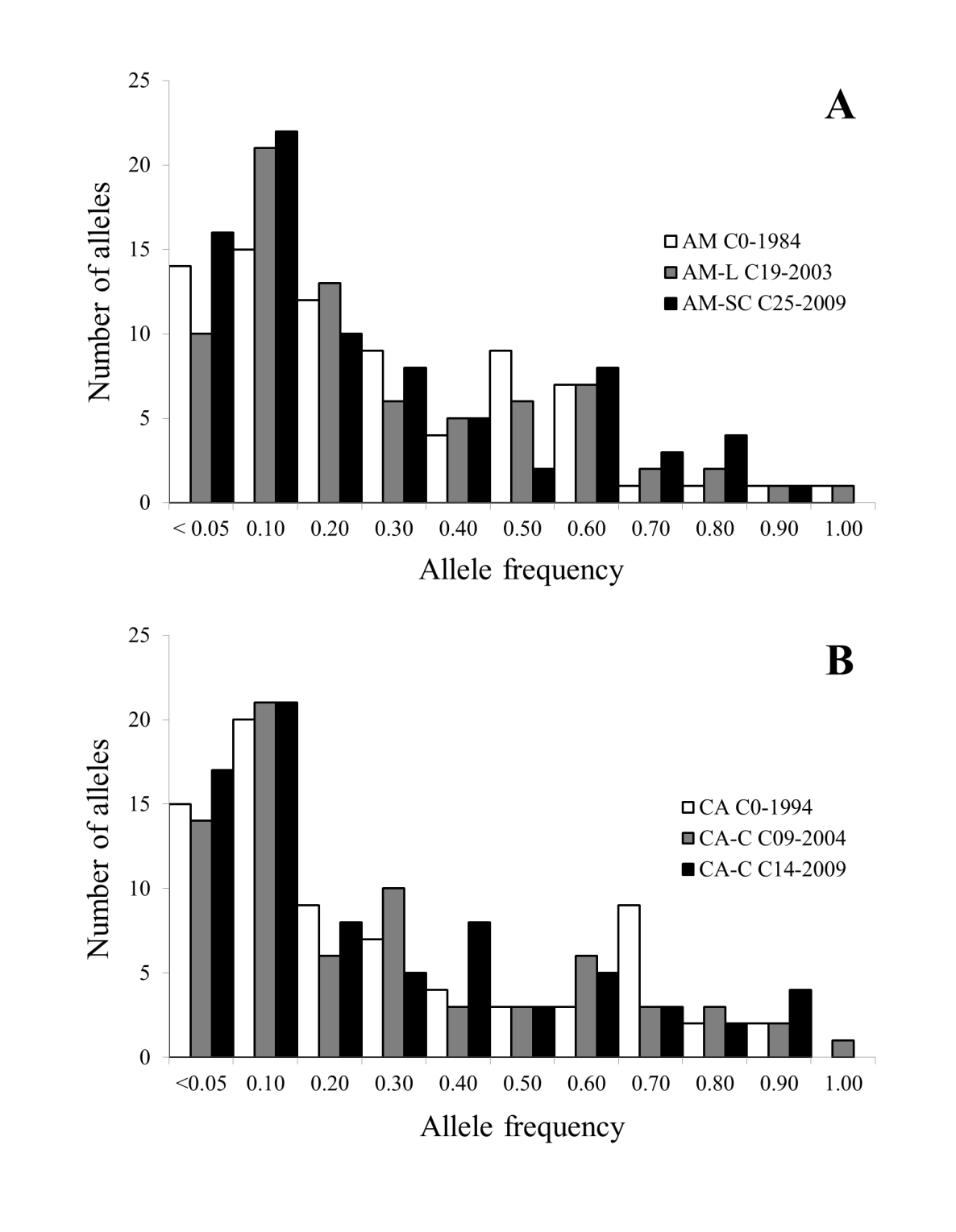


Figure S1. Allele frequency distribution in the Amiúdo and Castro Verde cycles: (A) Amiúdo initial population (AMC0-1984), selection cycle from the Lousada site (AM-LC19-2003) and selection cycle from the Serra do Carvalho site (AM-SCC25-2009); (B) Castro Verde initial population (CAC0-1994), and CA-CC09-2004 and CA-CC14-2009 selection cycles, both from the Coimbra site.

Literature cited

El-Sayed, A. M. 2014. The Pherobase: Database of Pheromones and Semiochemicals. Accessed April 12, 2011. http://www.pherobase.com.

FAO/IIASA/ISRIC/ISSCAS/JRC, 2012. Harmonized World Soil Database (version 1.2). FAO, Rome, Italy and IIASA, Laxenburg, Austria.

Instituto Português do Mar e da Atmosfera, I. P. (IPMA, I. P.). Climate Monitoring. Accessed February 2, 2017. https://www.ipma.pt/en/oclima/monitorizacao/
